# Regulation of PD-L1 Expression by SAHA-Mediated Histone Deacetylase Inhibition in Lung Cancer Cells

**DOI:** 10.3390/cancers17172919

**Published:** 2025-09-05

**Authors:** Umamaheswari Natarajan, Appu Rathinavelu

**Affiliations:** 1 Rumbaugh-Goodwin Institute for Cancer Research, Nova Southeastern University, Ft. Lauderdale, FL 33314, USA; un15@nova.edu; 2 Barry and Judy Silverman College of Pharmacy, Nova Southeastern University, Ft. Lauderdale, FL 33314, USA

**Keywords:** HDAC inhibitor, epigenetic alterations, PD-L1, cell cycle regulators, lung cancer

## Abstract

Background/Objectives: The effects of PD-L1 are mediated via its binding to the PD-1 receptor, which mediates the signals intracellularly to suppress T-cell responses. The expression levels of PD-L1 on cancer cells are an important indicator of immunosuppression and cause poor prognosis in several types of cancers. Therefore, the identification and characterization of mechanisms that regulate the expression of PD-L1 in cancer patients is very critical. Method: Our experiment was designed to determine the impact of histone deacetylase (HDAC) inhibitor on PD-L1 expression to reverse tumor-induced immunosuppression using H460 and HCC827 lung cancer cell lines. These cells were treated with the HDAC inhibitor suberoylanilide hydroxamic acid (SAHA). PD-L1 expression levels were assessed along with key regulatory proteins, including p53, p21, acetyl-histones, DNMT3B, MGMT, and trimethyl histones. Results: In our experiments, suberoylanilide hydroxamic acid (SAHA) was able to reduce the expression of PD-L1 by 60% in a dose-dependent manner. While the level of PD-L1 was significantly reduced, a concurrent increase in levels of p53, p21, and acetyl histone levels were observed in H460 and HCC827 cells following SAHA treatment. Furthermore, SAHA treatment was able to decrease the levels of DNMT3B, MGMT, and tri-methyl histones. It appears that the decrease in PD-L1 expression observed is solely because of p53 or p21WAF1/CIP1-mediated negative control on the transcription process. Conclusion: Our results suggest that SAHA can be used along with immune checkpoint inhibitors to potentiate the therapeutic outcomes in patients with excessive immunosuppression due to PD-L1 expression.

## Simple Summary

Immune evasion is a major challenge frequently seen during cancer therapy, in which the PD-L1 protein plays a key role by allowing cancer cells to suppress immune responses. High PD-L1 expression is associated with poor outcomes in several cancers, including lung cancer. This study explores how a drug called SAHA (suberoylanilide hydroxamic acid), which affects how genes are turned on and off, may help reduce the ability of lung cancer cells to evade the immune system. A key protein called PD-L1 (programmed death-ligand 1) helps cancer cells hide from immune attack, and high levels of PD-L1 are linked to worse outcomes in many cancers. Our results show that SAHA significantly reduces PD-L1 levels while simultaneously increasing the level of proteins involved in tumor suppression and cell cycle regulation. These findings suggest that SAHA may enhance immune recognition of cancer cells and support the potential strategy for combining epigenetic modulators with immunotherapy to overcome immune evasion and improve treatment outcomes in lung cancer. The study also observed a decrease in DNA methylation-related enzymes and histone modifications associated with gene silencing. Our results suggest that epigenetic modulation significantly influences PD-L1 expression and offers a potential strategy to enhance immune responses against cancer. Further research is needed to clarify the precise molecular mechanisms underlying these effects.

## 1. Introduction

Each year, a vast number of people die due to lung cancer than any other cancer, such as prostate, colorectal, pancreatic, liver, leukemia, esophageal, urinary bladder, brain, and breast cancer. Lung cancer accounts for the most number of cancer-related deaths among men (65,790) and women (59,280), making up almost 44% of the cancer-related deaths [1]. Non-small cell lung cancer (NSCLC) is the second most common form of lung cancer and the top leading cause of lung cancer-related deaths. As per the most recent statistics, about 80–85% of all lung cancers are NSCLC, and 13–15% are SCLC in the United States [2]. Despite enormous levels of progress in the areas of diagnosis, therapeutics, and prognosis of NSCLC, the overall treatment outcomes remain very poor for this type. Among the various advancements related to lung cancer treatment, immunotherapy has strongly emerged as a highly effective treatment option in recent years. At present, immune checkpoint (IC) blockade therapy is one of the most commonly used approaches for cancer immunotherapy. The PD-1/PD-L1 pathway is a well-studied immune checkpoint regulator that has been used to treat a variety of malignancies, including lung cancer.

Therapeutic antibodies that target PD-1 and its ligand PD-L1 have been approved for treating NSCLC, melanoma, Hodgkin’s lymphoma (HL), transitional cell carcinoma (TCC), renal cell carcinoma (RCC), breast cancer (BC), Merkel cell carcinoma (MCC), hepatocellular carcinoma (HCC), head and neck squamous cell carcinoma (HNSCC), gastric cancer (GC), and many other cancers [3,4]. The PD-L1 is expressed constitutively at high levels on both hematopoietic and non-hematopoietic cells. Examples of non-hematopoietic cells expressing PD-L1 at high levels include endothelial, epithelial, and muscle cells. A high-level expression of PD-L1 has been seen in various types of human cancers, including lung, bladder, colon, breast, ovary, kidney, cervix, melanoma, bone, glioblastoma, multiple myeloma, and T-cell lymphoma. Interestingly, in addition to binding to PD-1, PD-L1 interacts with the CTLA-4 (cytotoxic T-lymphocyte-associated protein 4), CD28, and CD80 (B7-1) T-cell markers, which influence T-cell function [5]. Moreover, the overexpression of PD-L1 on tumor cells is shown to correlate with poor treatment outcomes in most cancer types [6]. Therefore, several monoclonal antibodies have been developed for checkpoint rescue therapy to induce antitumor immunity by activating suppressed T-cells. In recent times, this therapeutic approach has revolutionized cancer immunotherapy, and as a result, immunotherapeutics have led to extraordinary increases in overall survival of patients, first with anti-CTLA-4 antibody and subsequently with anti-PD-L1 antibodies, in lung cancer, melanoma, and other malignancies [7,8]. The literature evidence clearly shows that, while treating advanced solid tumors, the therapeutic outcome of PD-1 pathway blockade is very well correlated with PD-L1 expression levels by the tumor cells and therefore stands as a prime model for targeted immunotherapies [7,8]. Consequently, innovative methods of treatment that employ new agents to block PD-1/PD-L1 or PD-1/PD-L2 interactions are on the rise, since this strategy has been yielding substantial benefits in multiple cancers [9].

In the last few decades, understanding genetic modulations has become an essential part of treating all types of cancers. More recently, epigenetic alterations in lung cancers were shown to be the anchorage of various genetic modifications, and therefore, epigenetic modifiers have also become novel therapeutic targets. Epigenetic alterations that can play a major role in the up-regulation of immune checkpoints (ICs) and their ligands at the transcriptomic level include histone modifications (acetylation, methylation, phosphorylation, adenylation, ubiquitination, and ADP ribosylation) and DNA methylations. In particular, acetylation of histone and DNA methylation are frequently found to be involved in the regulation of PD-L1 expression in cancer cells. Acetylation of lysine in histones is regulated by histone acetyltransferases (HATs), which is generally associated with an increase in transcription. Acetylation of histone can lead to an open chromatin structure in the promoter region that stimulates gene transcription. In addition, DNA methylations are significantly involved in causing epigenetic alterations that can impact immune responses and T-cell exhaustion. Therefore, epigenetic therapeutics, such as HDACIs (histone deacetylase inhibitors) and DNA methyltransferase (DNMT) inhibitors, can be used to alter the epigenetic abnormalities that are acquired during the disease progression [10,11].

Among the various types of epigenetic alterations that can influence gene expression, histone modifications are suspected to be the most important in regulating PD-L1 gene expression [12]. For example, the histone acetylation of the promoter region of the PD-L1 gene is essential for the regulation of ICs. HDACIs can inhibit HDAC-mediated deacetylation, leading to hyper-acetylation of histones and the re-expression of epigenetically silenced genes. HDACIs can activate gene expression by altering the transcription of various proteins via inducing histone acetylations, transcription factors, and a vast array of proteins. Therefore, HDACIs, which are a new class of small-molecular therapeutics, are known to induce the inhibition of pathways that are involved in angiogenesis, cell cycle arrest, and cell death. HDACIs can induce a wide range of immunological alterations and transient modifications of gene expression without affecting DNA sequences [13,14]. So far, various clinical studies have demonstrated that blocking the PD-1/PD-L1 pathway, which is a vital regulator of T-cell activity, could boost the antitumor immune response and thereby inhibit the growth of the tumors. A comprehensive understanding of the molecular and cellular interactions between PD-1 and PD-L1, and the extent to which this pathway influences immune regulation across diverse cancer types is critical for developing newer treatment strategies. To address this, we analyzed PD-L1 expression patterns in various cancer cell lines to gain further insight into its role in immune escape. In addition, we explored the impact of the HDACI on the expression levels of PD-L1 using the H460 and HCC827 lung cancer cells. These two lung cancer cell lines were selected for this study due to their high basal level expression of PD-L1, as well as their distinct p53 and p21 profiles. H460 cells express wild-type p53 with relatively higher basal p21 levels, whereas HCC827 cells harbor mutant p53. This combination allows for the evaluation of SAHA’s effects on PD-L1 regulation and immune checkpoint pathways across different molecular status and contexts in lung cancer. In this regard, SAHA was suspected to decrease the PD-L1 expression either directly or indirectly in cancer cells [13,14]. Reduced expression of PD-L1 was expected to be associated with elevated antitumor efficacy. Therefore, our current study aims to assess the mechanisms that may regulate the PD-L1 expression during HDAC inhibition in lung cancer cells.

## 2. Materials and Methods

### 2.1. Cell Lines and Reagents

The H460 and HCC827 cells (human non-small lung cancer cell lines) were purchased from ATCC (American Type Culture Collection, Manassas, VA, USA). Both cells were cultured within RPMI -1640 medium, supplemented with 10% fetal bovine serum (FBS), 1% L-glutamine, and 1% penicillin/streptomycin. All cells were maintained with 5% CO_2_ and 95% air at 37 °C. All cell lines used in this study were routinely tested for mycoplasma contamination to ensure the reliability and reproducibility of our experimental results. SAHA (HADC inhibitor) was purchased from Selleckchem (Houston, TX, USA). Most of the primary antibodies were purchased from Cell Signaling Technology (Danvers, MA, USA). The PD-L1 antibody was purchased from R&D Systems (Minneapolis, MN, USA). MDM2-specific antibody was purchased from Santa Cruz Biotechnology, Inc. (Dallas, TX, USA) Table 1. The β-actin-specific antibody and the secondary antibodies (anti-rabbit and anti-mouse) conjugated to horseradish peroxidase (HRP) were obtained from Sigma Aldrich (St. Louis, MO, USA). Western Blotting Detection Reagents-KPL LumiGlo Reserve chemiluminescent substrate was obtained from Sera Care Life Sciences (Milford, MA, USA).

### 2.2. Western Blotting

The expression levels of epigenetic modifications and cell cycle markers were measured using Western blotting. Briefly, drug-treated H460 and HCC827 cells were lysed on ice using 1X RIPA cell lysis buffer containing protease inhibitor cocktail, PMSF (phenyl methyl sulfonyl fluoride), and sodium orthovanadate. Total protein concentrations were measured using the bicinchoninic acid (BCA) assay kit (Thermo Fisher Scientific Inc., Waltham, MA, USA) according to the manufacturer’s instructions. Equal amounts (25 µg) of total protein were resolved on 5–15% SDS-PAGE and blotted onto nitrocellulose membranes (Amersham Biosciences, Little Chalfont, UK). The membranes were sliced into two or three portions of different molecular weight ranges and blocked with 5% (*w*/*v*) non-fat, dry skim milk and incubated individually with the primary antibodies overnight with gentle shaking at 4 °C. The membranes were incubated with corresponding HRP-conjugated (Invitrogen, Carlsbad, CA, USA) secondary antibodies at room temperature for 1 h. Finally, signals were detected by using the KPL LumiGlo Reserve chemiluminescent substrate, and the images were captured using a UVP image analyzer (EC3 Chemi HR 410 imaging system). The intensity of the protein band was determined by densitometric measurement using the ImageJ 1.53e software (National Institute of Health, Bethesda, MD, USA). Target protein intensities were validated using the densitometry analysis of the corresponding β-actin loading control to alleviate variations that may occur due to protein loading or transfer efficiency.

### 2.3. Immunostaining for Acetylation of Histones

We utilized an immunofluorescence staining method that allowed for the identification of p21, acetyl histones, and PD-L1. The 2D (monolayer) lung cancer cells were seeded at a density of 5 × 10^4^ cells/well and treated with 7.5 µM of SAHA for 24 h. After incubation, the treated cells were first fixed with 10% formalin for 15 min at room temperature and washed three times with - phosphate-buffered saline (PBS). Monolayer cells were permeabilized with 0.5% of Triton-X in PBS at room temperature for 10–15 min. The permeabilized cell samples were blocked with 3% BSA (bovine serum albumin) in PBS with 0.5% Triton X-100 at room temperature for 1 h. Following permeabilization, the 2D monolayers were washed with PBS and incubated with primary antibodies specific to acetyl histones (Ac-Histone-H2A, Ac-Histone-H2B, Ac-Histone-H3, and Ac-Histone-H4), which were diluted 1:100 in PBS with 3% BSA and 0.1% Triton X-100 and incubated overnight at 4 °C. After the incubation periods, the 2D cells were washed 3 times with PBST and then incubated with the Alexa-Fluor^®^ 488 green (rabbit) conjugated secondary antibodies at room temperature for 1 h. The cells were washed 3 times with PBS before the immunofluorescence images were acquired at 10X magnification using a DMI3000 B Leica fluorescence microscope.

### 2.4. RNA Extraction

Total RNA was extracted from control and SAHA-treated H460 and HCC827 cells. The RNA isolation was performed using the RNeasy-mini kit according to the manufacturer’s protocol (Qiagen, Valencia, CA, USA). The purity and concentration of total RNA were determined by measuring the ratio of absorbance at 260/280 and 260/230 nm.

### 2.5. cDNA Synthesis Using the RT^2^ First Strand Kit

The cDNA was synthesized using total RNA as the templates isolated from control and SAHA-treated H460 and HCC827 cells with the RT^2^-first strand kit as per the manufacturer’s protocol (Qiagen, Valencia, CA, USA). The genomic DNA elimination mix for each RNA sample consists of 500 ng of RNA, 2 µL of Buffer GE, and 6 µL of water. The genomic DNA elimination mix was incubated for 5 min at 42 °C and then placed immediately on ice for at least 1 min. The reverse transcriptase mix of 10 µL was added to each tube containing 10 µL of genomic elimination mix, and the mixer was pipetted up and down. The mixer was incubated at 42 °C precisely for 15 min. The reaction was stopped by incubating the tubes at 95 °C for 5 min. Finally, 91 µL of RNase-free water was added to each reaction. The cDNA was used for the RT^2^ Profiler PCR Array analysis.

### 2.6. Human Epigenetic Chromatin Modification Enzymes RT2 Profiler PCR Array

Gene expression profiling using the cDNA synthesized by the method described above was conducted using the Human Epigenetic Chromatin Modification Enzymes RT^2^ profiler PCR array (Catalog #330231 PAHS-085Z, Qiagen, SABiosciences, San Diego, CA, USA). This array was designed to profile the pathway expression analysis of 84 key genes known or predicted to epigenetically modify genomic DNA and histones to regulate chromatin accessibility. Quantitative reverse transcription PCR (qRT-PCR) was conducted using the ABI StepOnePlus Real-time PCR system (Applied Biosystems, Foster City, CA, USA) following the instructions of the microarray manufacturer. Relative quantification of gene expression was determined using the double delta Ct (∆∆Ct) method. The heat map, fold changes of gene expression, and scatterplot were analyzed and generated by using the RT^2^ PCR array data analysis web portal: https://dataanalysis2.qiagen.com/pcr (accessed on 21 October 2024). The heat map represents the lowest and highest gene expression compared to the reference gene samples. Genes of SAHA-treated groups that had fold changes of more than 2 in expression levels against control groups were considered significant.

### 2.7. Biological Pathway Analysis Using STRING

Differentially expressed genes were analyzed using STRING version 12.0, https://string-db.org/cgi/input.pl (accessed on 27 May 2025) which is an online research tool for collating gene interactions. STRING is a database of known and predicted protein-protein interactions. The interactions include direct (physical) and indirect (functional) associations, which stem from computational predictions, and interactions aggregated from other (primary) databases. The STRING database integrates collective biological knowledge through text mining, data mining, data comparison, and computational prediction. This tool was used to view the associations of the differentially expressed genes in H460 and HCC827 cells that were showing significant changes in the PD-L1 expression.

### 2.8. Statistical Analysis

For all in vitro experiments, statistical analyses were performed using one-way analysis of variance (ANOVA). Data are presented for the Western blots are mean ± SD from at least three biological replicates. Significance is indicated as * *p* < 0.05, ** *p* < 0.01, and *** *p* < 0.001. For the RT^2^ Profiler Array Analysis the fold change and the *p*-values were calculated using the online analysis tool GeneGlobe provided by Qiagen Inc. Results with fold change greater than 2 and *p*-value < 0.05 were considered statistically significant.

## 3. Results

### 3.1. Expression of PD-L1 in Different Lung Cancer Cells and the Effects of SAHA

**Figure 1:**
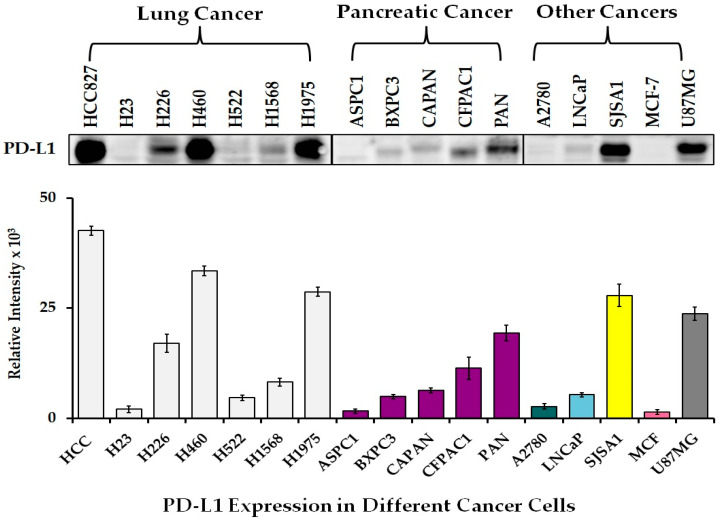
Representative Western blot images showing the levels of PD-L1 in different cancer cell lines. All light-gray shaded bars are lung cancer cell lines and purple shaded are pancreatic cancer cell lines. (uncropped Western blot Supplementary Figure S1).

**Figure 2:**
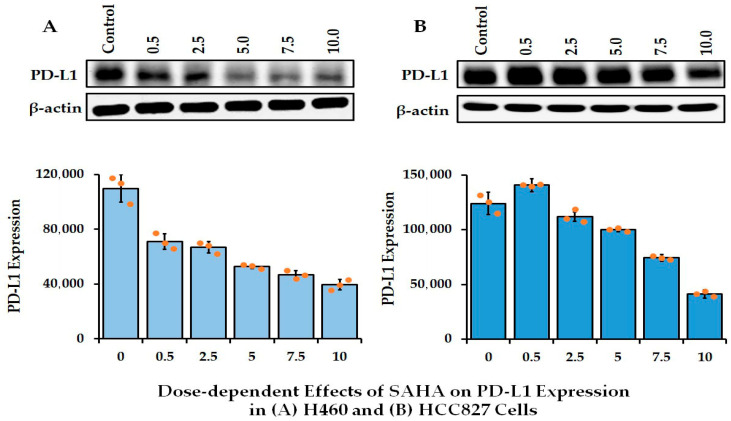
Representative Western blot images showing the changes in the levels of PD-L1 protein expression in lung cancer cells after SAHA treatments. Dose-dependent response of PD-L1 in (**A**) H460 and (**B**) HCC827 cells after SAHA (0–10 μM) treatment. The bottom panel represents the band intensity of the PD-L1 proteins normalized to that of β-actin using ImageJ software. The bar graph presents the mean PD-L1 expression at each SAHA dose with standard deviation error bars, while the scatter points indicate the distribution of the individual replicates (uncropped Western blot Supplementary Figure S1).

In our previous publication, we reported the expression levels of PD-L1 in seven different types of lung cancer cells (HCC827, H23, H226, H460, H522, H1568, and H1975), which confirmed that the most significant level of expression was occurring in HCC827, H460, and H1975 cells [12,13]. Among the seven lung cancer cells tested, the highest expression of PD-L1 was found in HCC827 cells. Both H460 and H1975 cells also showed PD-L1 expression, which was slightly lower than what was seen in HCC827 cells. On the other hand, in H23, H1568, and H522 cells, PD-L1 expression was shown to be at a lower level [12,13]. In addition, we analyzed the level of PD-L1 expression in other cancers, including pancreatic (ASPC1, BXPC3, CAPAN, CFPAC1, and PAN), ovarian (A2780), prostate (LNCaP), breast (MCF-7), osteosarcoma (SJSA1), and glioblastoma (U87) cells. Among the various cells tested, we observed a high level of PD-L1 expression in SJSA1 and U87 cell lines compared to others (Figure 1). Therefore, the regulation of the PD-L1 level was further assessed in this study using H460 and HCC827 lung cancer cells. When HCC827 and H460 cells were treated with different concentrations of SAHA, ranging from 0.5 µM to 10 µM, for 24 h, the level of PD-L1 showed a steady decrease, with the maximum decrease observed in cells treated with 10 µM concentration (Figure 2A,B). Thus, the level of PD-L1 expression in H460 and HCC827 cells decreased in a dose-dependent manner.

### 3.2. Expression of Cell Cycle Regulators

**Figure 3:**
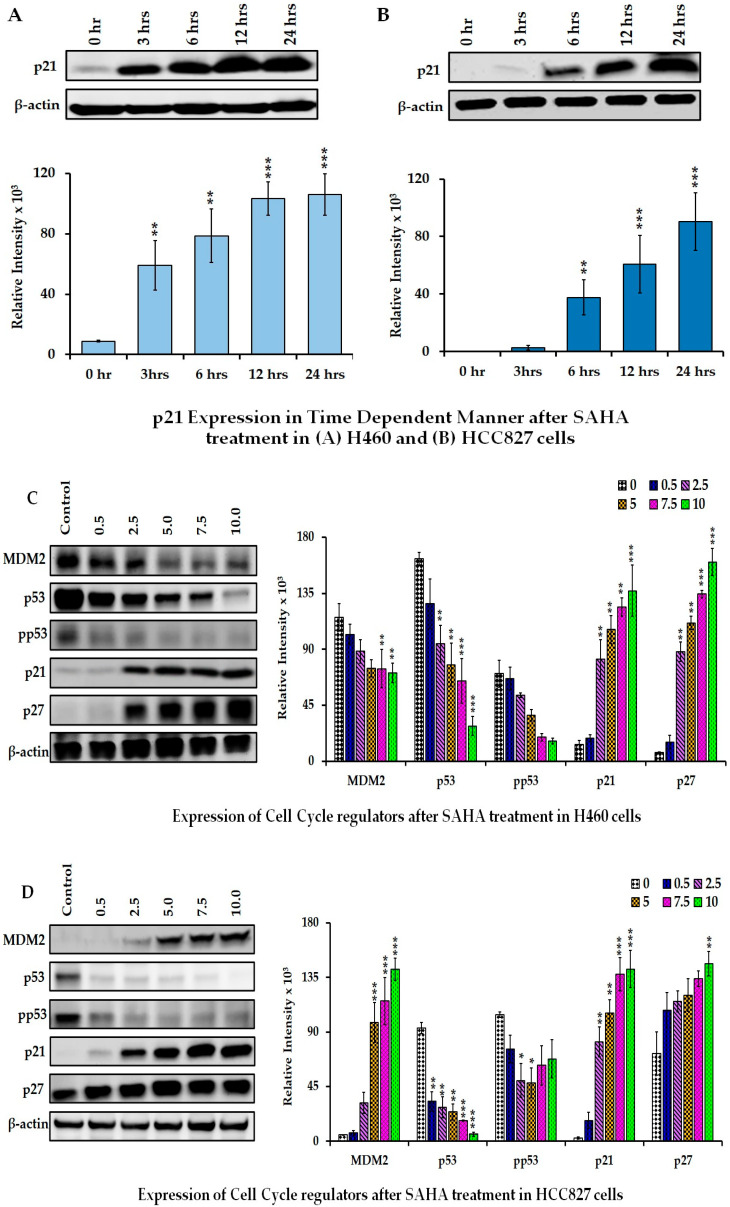
Representative Western blot images showing the changes in the levels of cell cycle-related proteins after SAHA (0–10 μM) treatments. Time-dependent effect of p21 expression after SAHA (7.5 µM) treatment in (**A**) H460 and (**B**) HCC827 cells. Dose-dependent effect of SAHA on p21 and other cell-cycle-related proteins in H460 (**C**) HCC827 (**D**) cells are shown. The right panel shows the band intensity of cell cycle proteins validated with the intensities of β-actin bands using ImageJ software. Data are presented as means ± SD from at least three independent experiments. Statistical significance is indicated as * *p* < 0.05, ** *p* < 0.01, and *** *p* < 0.001 compared with the control group. (uncropped Western blot Supplementary Figure S1).

Once the dose -effects of SAHA on PD-L1 expression were confirmed, we analyzed the SAHA-treated cell samples for the p21, p27, p53, CDK4, CDK6, pRB, STAT3, and phospho-STAT3 levels. Among the HDACI-responsive genes, p21WAF1/CIP1 was one of the most commonly altered after drug treatments. To further investigate the regulation of p21WAF1/CIP1 expression level, H460 and HCC827 cells were incubated with SAHA for different time points and concentrations (Figure 3A–D). During time- and dose-dependent studies, the experiments were started in the morning to complete 12 h and 24 h treatments without interruption. In addition to p21WAF1/CIP1 levels, we also analyzed the expression levels of the other cell cycle regulatory proteins (MDM2, p53, phospho-p53, p21WAF1/CIP1 and p27Kip1) after SAHA treatment in H460 and HCC827 cancer cells. As shown in Figure 3A,C, we found that SAHA increased p21WAF1/CIP1 and p27Kip1 protein levels in both lung cancer cell lines in a dose-dependent manner. Similarly, the level of p27 was also significantly elevated in H460 cells starting from 2.5 µM SAHA treatment. However, in HCC827 cells there was only a slight elevation of p27, which was not as robust as the elevation observed in H460 cells (Figure 3B,D). Furthermore, we observed that SAHA caused significant decreases in the levels of MDM2, p53, and phospho-p53 (Figure 3C). The decrease in gene expression in H460 and HCC827 cells appears to be very similar even though the p53 status for these cells are different. These results suggested that, when p21 levels were elevated following SAHA treatment, the same concentrations did not elevate the p53 levels; rather, there was a slight decrease in p53 levels in both H460 and HCC827 cells.

### 3.3. Effect of SAHA on STAT3, CDK4, CDK6, NF-κB, and pRB Levels

**Figure 4:**
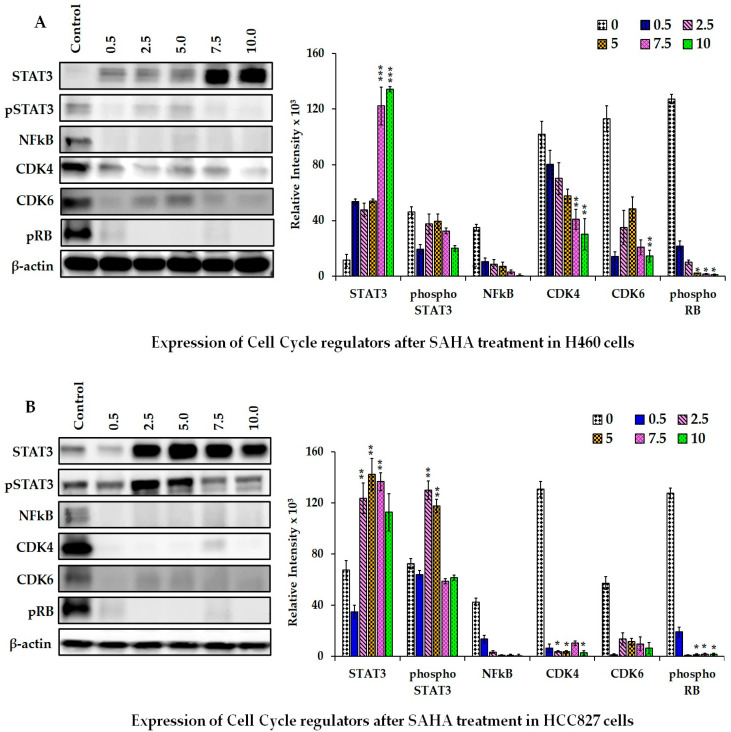
Representative Western blot images showing the changes in the levels of STAT3, phospho-STAT3, CDK4, CDK6, NFκB, and pRB protein levels after SAHA (0–10 μM) treatments in H460 cells (**A**) and HCC827 cells (**B**). The right panel represents the band intensity of the cell cycle proteins normalized to that of β-actin using ImageJ software. The data are presented as means ± SD from at least three independent experiments. The level of significance is indicated as * *p* < 0.05, ** *p* < 0.01, and *** *p* < 0.001 compared to control. (uncropped Western blot Supplementary Figure S1).

SAHA treatment was able to significantly up-regulate the level of STAT3 starting from 0.5 µM concentration in H460 lung cancer cells, and the maximum elevation was seen with 7.5 and 10 µM concentrations (Figure 4A,B). In HCC827 cells also, STAT3 levels were elevated following SAHA treatment; however, the elevations started to appear following treatments with 2.5 µM concentrations of SAHA in HCC827 cells. In addition to analyzing p53, p21, and p27 levels, we also analyzed the status of STAT3, phospho-STAT3, NF-κB, CDK4, CDK6, and pRB levels following HDAC inhibition with SAHA. Furthermore, the PD-L1 level was also found to be significantly decreased after SAHA treatment. Interestingly, similar to the decrease observed with PD-L1 levels, different concentrations of SAHA treatment were decreasing the levels of CDK4, CDK6, and phospho RB in H460 and HCC827 cells after 24 h. Reduced levels of CDK4, CDK6, and pRB coincided with the elevated levels of p21 after SAHA treatment in lung cancer cells confirmed cell cycle arrest, as shown in Western blot analysis (Figure 4A,B).

### 3.4. The Effect of SAHA on the Levels of Acetylated Histones

**Figure 5:**
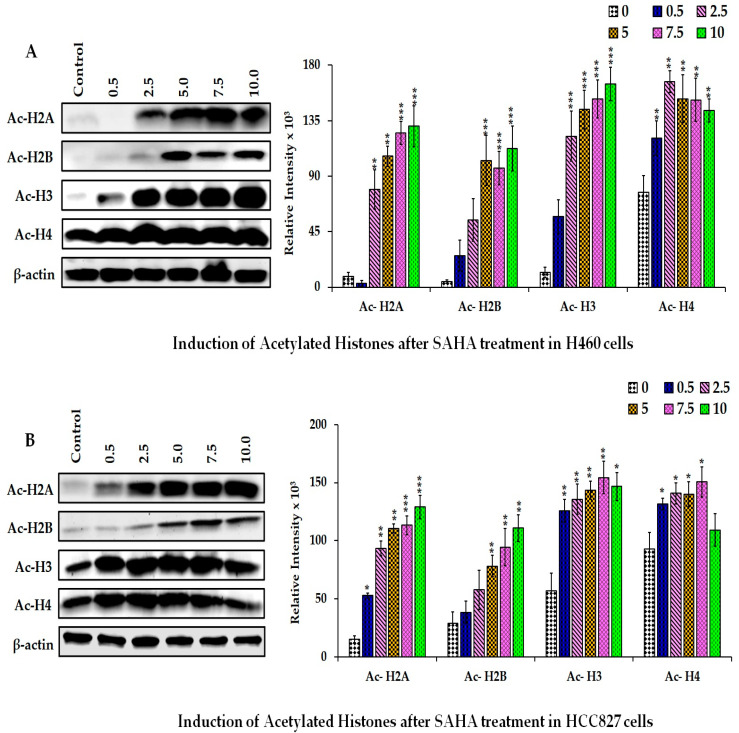
Representative Western blot images showing the changes in the levels of acetylation of histone-related proteins in H460 (**A**) and HCC827 (**B**) cells after treatments with different concentrations (0–10 μM) of SAHA. The right panel represents the band intensity of the cell cycle proteins normalized to that of β-actin using ImageJ software. The data are presented as means ± SD from at least three independent experiments. The level of significance is indicated as * *p* < 0.05, ** *p* < 0.01, and *** *p* < 0.001 compared to control. (uncropped Western blot Supplementary Figure S1).

**Figure 6:**
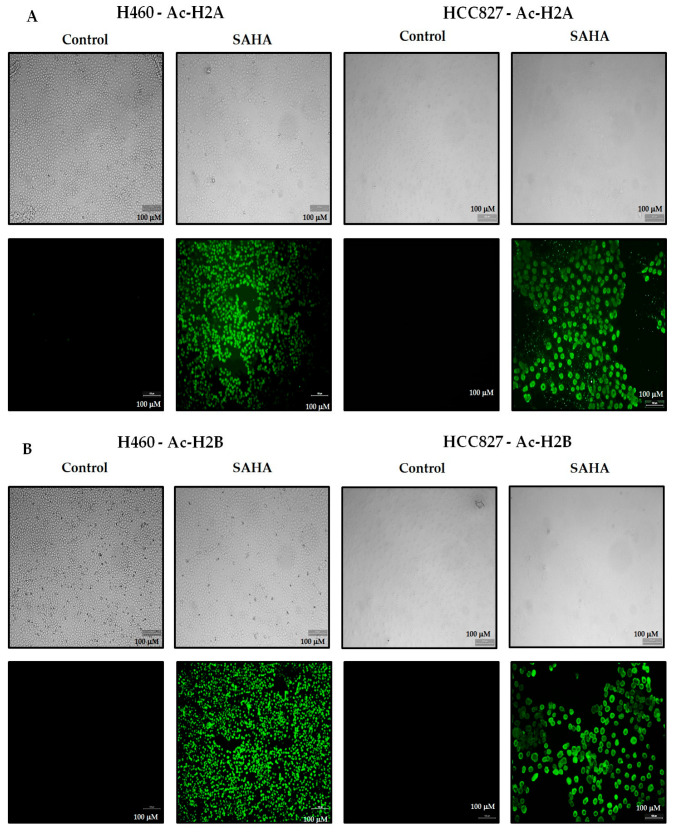

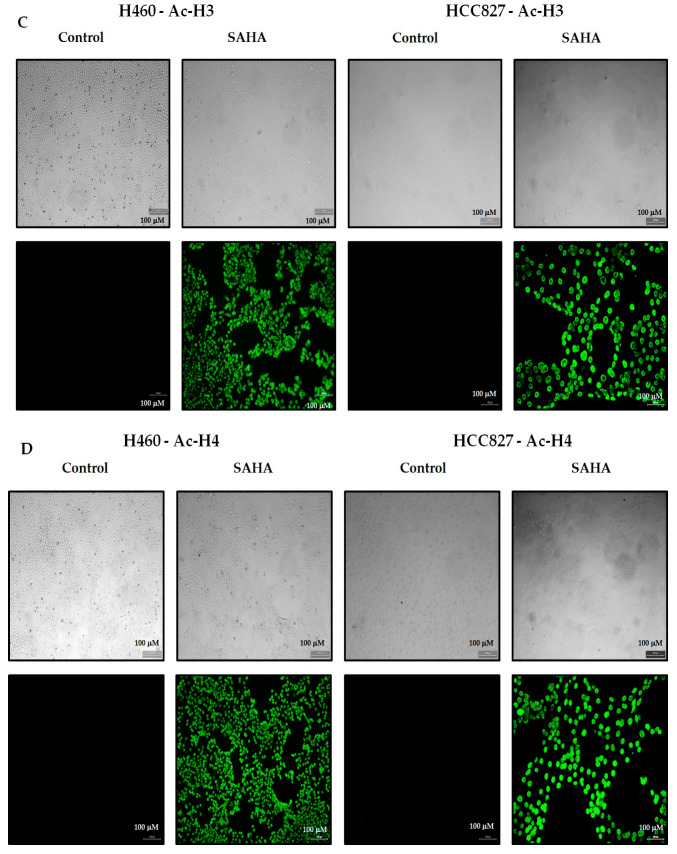
H460 and HCC827 cells grown as a monolayer (2D) stained for acetylated histones. Upper panels: light microscopic images of lung cancer cells following SAHA treatment (7.5 µM). Lower panels: immunocytochemical detection of Ac-H2A (**A**), Ac-H2B (**B**), Ac-H3 (**C**), and Ac-H4 (**D**) visualized using Alexa Fluor^®^ 488 and the images with green fluorescence were captured with Leica DMI3000 B microscope at 10× magnification. Scale bars = 100 µM.

Since the effect of SAHA is primarily mediated through the inhibition of HDACs, we analyzed the status of Ac-H2A, Ac-H2B, Ac-H3, and Ac-H4 in H40 and HCC827 cells with different concentrations of SAHA treatment. The levels of Ac-H2A, Ac-H2B, and Ac-H3 were significantly elevated in the H460 cells in a dose-dependent manner with a maximum elevation at 10 µM concentration of SAHA (Figure 5A,B). Interestingly, the Ac-H3 showed the maximum elevation starting from 2.5 µM of SAHA treatment. However, the elevation of Ac-H4 was not as robust as Ac-H3. In HCC827 cells, the levels of all four acetylated histones (Ac-H2A, Ac-H2B, Ac-H3, and Ac-H4) were also elevated but displayed differences in their dose-related responses. Both Ac-H2A and Ac-H2B showed a steady increase up to 10 µM concentration, while Ac-H3 and Ac-H4 showed maximum elevation around 2.5 µM concentration of SAHA treatment, which was significantly decreasing below the control levels at 10 µM concentration (Figure 5A,B). Among the four acetylated histones analyzed, the basal level as well as the elevated levels of Ac-H2B were consistently lower compared to Ac-H2A, Ac-H3, and Ac-H4. The changes were confirmed through the immunocytochemistry (ICC) analysis in both H460 and HCC827 lung cancer cells using specific antibodies (Figure 6A–D). The upper panels in Figure 6 show the light microscopic images of cells after SAHA treatment, showing the shape and size and some of the morphological features. The lower panels display the fluorescence images indicating the acetylation status of the corresponding histones. This analysis demonstrates that SAHA differentially modulates histone acetylation patterns in these lung cancer cell lines, suggesting possible effects on chromatin remodeling, chromatin access, and consequently gene regulation.

### 3.5. The Effect of SAHA on the Levels of DNMTs and Methylated Histones

**Figure 7:**
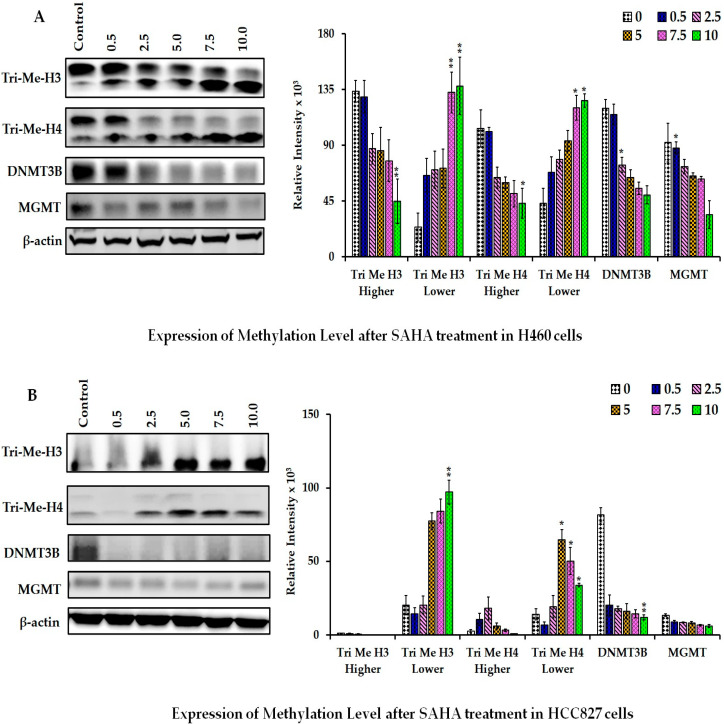
Representative Western blot images showing the changes in the levels of DNA and histone methylation-related proteins after different concentrations of SAHA (0–10 μM) treatments. (**A**) H460 and (**B**) HCC827 cells. The right panel represents the band intensity of the cell cycle proteins normalized to that of β-actin using ImageJ software. The data are presented as means ± SD from at least three independent experiments. The level of significance is indicated as * *p* < 0.05 and ** *p* < 0.01 compared to control. (uncropped Western blot Supplementary Figure S1).

In our study, a few additional biomarkers were examined to elucidate the patterns and impacted signaling pathways following HDACI-induced hypomethylation in both lung cancer cells. We analyzed the levels of trimethyl H3 and trimethyl H4 after treating the cells with SAHA using Western blot analysis. As shown in Figure 7A,B, the treatment of SAHA in H460 and HCC827 cells showed a significant decrease in Me-H3 and Me-H4 levels (Figure 7A,B) after 24 h of treatment with SAHA in both H460 and HCC827 cell lines. Since different DNA methyltransferase (DNMT) family members are responsible for either de novo DNA methylation or for the maintenance of DNA methylations, we analyzed the levels of DNMT3B, which is one of the important methyltransferases, after treating the cells with SAHA. Interestingly, the HDACI treatment led to significant decreases in tri-methyl histones H3 and H4, DNMT3b, and MGMT levels.

### 3.6. Identification of Differential Gene Expression in Lung Cancer Cells Using the Heat Map Generated with the RT^2^ Profiler PCR Array Data

**Figure 8:**
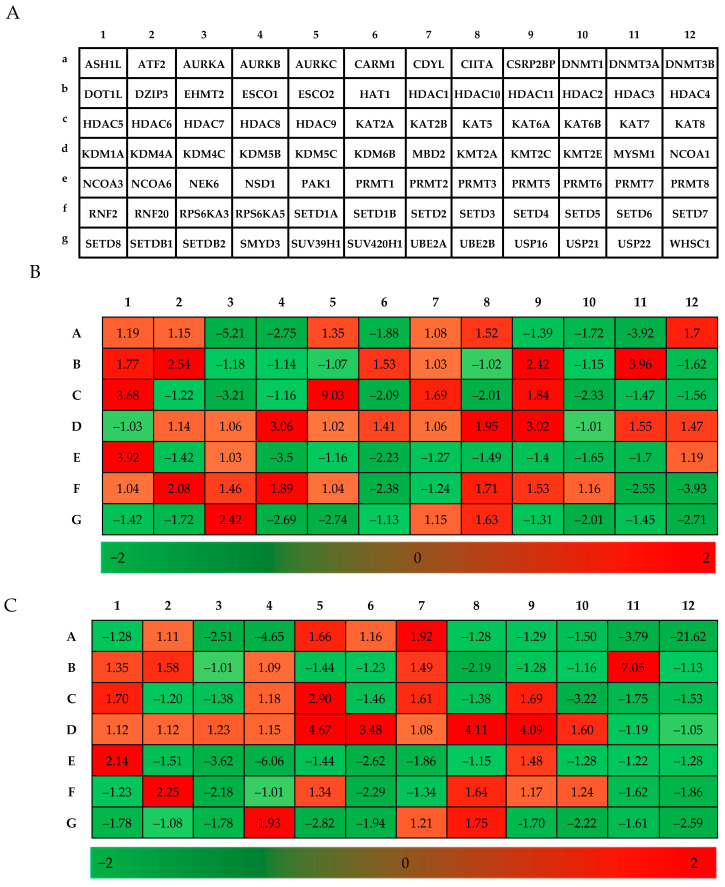
Human epigenetic chromatin modification enzymes gene used in RT^2^ profiler PCR array experiments. (**A**) Layout of the human epigenetic chromatin modification enzymes for the RT^2^ profiler PCR array. (**B**,**C**) Heat map showing differentially expressed genes after SAHA treatment in H460 and HCC827 cells, respectively. The red and green colors represent high- and low-level expression, respectively.

The heat map created for the differential expression of the genes shows the genes that were significantly altered in SAHA-treated cells compared to control (Figure 8A–C). In order to get an overview of the pathways impacted by SAHA treatment, we used the RT^2^ profiler PCR array data to create heat map, which showed that the expression of 11 and 8 genes were up-regulated by >2-fold in H460 and HCC827 cells, respectively. On the other hand, 16 and 14 genes were down-regulated <2-fold in SAHA-treated H460 HCC827 cells, respectively, compared to the untreated control cells (Table 2A,B, and Figure 8A,B). Among the deacetylation- and methylation-related genes, HDAC3, HDAC9, NCOA3, SETD3, SETD4, SETD5, KMT2A, KMT2C, and MBD2 were found to be significantly up-regulated in both H460 and HCC827 cells. Interestingly, KMD4A, KMD4C, KMD5B, KMD5C, and KMD6B were also up-regulated in both cell lines following SAHA treatment. Members of the SETD2 family typically methylate histone H3 Lys9 (H3K9), which is an epigenetic mark associated with gene silencing [15]. Similarly, KMT2A and KMT2B, which encode histone–lysine N-methyltransferases 2A, also known as acute lymphoblastic leukemia 1 or mixed-lineage leukemia protein (MLL1), were also elevated in both cell lines. However, the ASH1L gene, also known as the KMT2H gene that codes for the synthesis of lysine methyltransferase 2H was up-regulated only in H460 cells and not in HCC. In addition, the RNF20 gene, which codes for the E3 ubiquitin-protein ligase BRE1A, along with UBE2A and UBE2B, which are ubiquitin-conjugating enzymes E2A and E2B, respectively, were significantly up-regulated in both cell lines.

### 3.7. Network Analysis of Differentially Expressed Genes Using STRING

**Figure 9:**
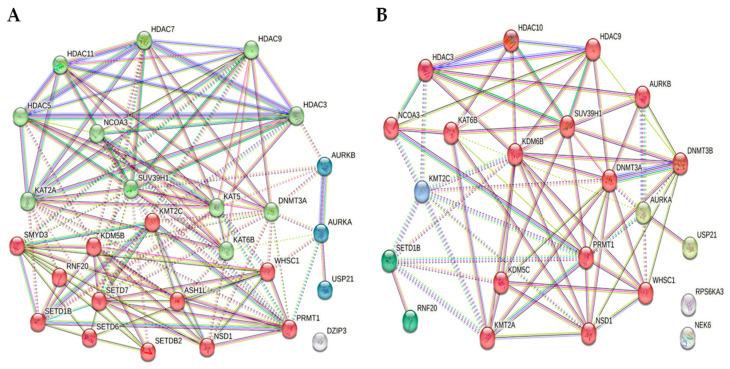
(**A**) Network analysis of the H460 protein–protein interaction (PPI) for the top 26 differentially regulated genes. (**B**) Network analysis of the HCC827 protein–protein interaction for the top 20 differentially regulated genes. Circles represent the genes, and the connecting lines represent the number of interactions between them. The colors of the nodes are clusters as indicated in Table 3. The color of the edges are as described in the Network section of the Getting Started page of String Help (https://string-db.org/help/getting_started/, accessed on 16 July 2025).

To identify the hub genes from the interaction network, a hybrid centrality measure method was employed. In H460 cells, the PPI network was created using the top 26 differentially regulated gene clusters as indicated in Figure 9A and Table 3A. Similarly, the PPI network created using the top 20 differentially regulated genes from HCC 827 cells is shown in Figure 9B and Table 3B. The PPI network constructed using STRING yielded three clusters, with Cluster 1 showing interactions among 12 proteins and Cluster 2 showing interactions among 11 proteins in H460 cells. Similarly, the PPI network constructed for HCC 827 cells yielded four clusters, with Cluster 1 showing interactions among 15 proteins. The proteins in the PPI network of both cell lines may be negatively or positively correlated to PD-L1 expression.

## 4. Discussion

Epigenetic alterations such as histone modification and DNA methylation have been investigated for quite some time in various cancers, including lung cancers. So far, it has been well established that both HATs and HDACs are involved in regulating cell growth, differentiation, cell cycle arrest, and cell death by transforming heterochromatin of the DNA to euchromatin. Heterochromatin is a highly condensed and transcriptionally silent form, whereas euchromatin is a less condensed and more relaxed structure; therefore, it can be easily transcribed [16]. Lately, HDACIs have been found to be associated with an increase in histone acetylation and reversal of tumorigenesis and cell proliferative mechanisms. The use of HDAC inhibitors for treating different cancers, including lung cancers, has been reported by various investigators with significant outcomes [17]. In this regard, several studies have shown that SAHA treatment can increase the levels of acetylated histones such as Ac-H2A, Ac-H2B, Ac-H3, and Ac-H4, compared to the controls, which are known to contribute to the regulation of both the transcriptional and post-transcriptional mechanisms that can deactivate proliferation of cancer cells [10,11,12,13,14,18]. However, following SAHA treatment, H3 and H4 acetylation was not elevated in HCC827 cells. The differential Ac-H3 responses in H460 versus HCC827 appears to be due to p53 status. H460 cells have wild-type p53; in contrast, HCC827 cells harbor mutant p53. It has been reported that cells with mutant p53 are unable to show elevation in H3 acetylation, unlike cells with wild-type p53 [19]. However, at the highest dose of SAHA, there was a decrease in H3 acetylation, which could be due to the decrease in the levels of histones caused by sustained epigenetic alterations.

While acquiring growth and proliferative ability, cancer cells can evade attacks from the immune system through various mechanisms. Many recent studies have revealed that cancers can escape from the immune system through the down-regulation of antigen expression on tumor cells, a decrease in the number of lymphocytes, suppression of cytotoxic T-cells, etc. [20]. Among these mechanisms, the programmed death ligand-1/programmed death-1 receptor (PD-L1/PD-1) signaling pathway is an important component of tumor immunosuppression, which can inhibit the activation of T lymphocytes and create an immune tolerance towards tumor cells, leading to the escape of cancer cells from the immune system [21]. Different types of cancer cells express PD-L1 on their surface, which is critical for triggering immunosuppression and helping the cancer cells to survive by evading the immune attack. In this regard, the inhibition of PD-L1 activity was validated to enhance tumor cell attack under both in vivo and in vitro conditions. However, the principle of specific mechanisms stemming from epigenetic alterations that can influence PD-L1 expression and impact on immunosuppression is yet to be established. Epigenetic modifiers such as SAHA are used to treat different types of cancers because of their broad-spectrum activity towards different classes of HDACs. Similar to our interest, the role of epigenetic modifiers in sensitizing cancers to different therapeutics [22], in particular the use of combination treatment to augment PD-L1 targeted immunotherapies [23], has been in consideration for quite some time in many other laboratories. In this connection, previous research work in our laboratory also has shown that SAHA could produce significant antitumor effects in solid tumors such as breast, prostate, and ovarian cancers [24,25,26,27,28,29]. Beyond the direct repression of cancer cell growth, SAHA has also been reported to have the ability to regulate the immune system [12,13,14,30,31]. Furthermore, our laboratory had reported a high-level expression of PD-L1 in different types of lung cancer, pancreatic, osteosarcoma, and glioblastoma cells. Interestingly, after treatment with SAHA, the level of PD-L1 expression was down-regulated in many of these cancer cells, particularly in H460 and HCC827 cells [14,25,26]. This effect of SAHA on PD-L1 expression appears to occur through two mechanisms. First and foremost, the most direct mechanism following SAHA treatment is through increasing the acetylation of H2A, H2B, H3, and H4 levels in targeted cancer cells. However, it appears that the decrease in the expression of PD-L1 observed in lung cancer cells may not be solely as a result of the acetylation of histone in the promoter region of PD-L1 genes. The promoter region of the PD-L1 gene is reported to contain 18 CpG islands, and the levels of H3 acetylation were reported to be significantly altered in drug-resistant cancer cells [32]. It has been previously reported that PD-L1 expression can increase with H3 acetylation [24]. However, in our experiments, the level of PD-L1 decreased even when there was an increase in H3 acetylation (Figure 5A,B). Hence, we suspect that other mechanisms involving p21 or p27 may be more important in addition to the acetylation of histones in regulating PD-L1 levels. In this study, HDAC3 was also reported to correlate with the suppression of PD-L1 expression in cancer cells through the modification of H3 acetylation [33]. In essence, this study concluded that an aberrant expression of HDAC3 reversed H3 acetylation in the PD-L1 promoter region and decreased PD-L1 expression in drug-resistant cancer cells. Thus, some of the previous results have indicated that decreasing histone H3 acetylation of the PD-L1 promoter region would cause a decrease in its expression. However, we saw elevation of H3 and H4 acetylation following SAHA treatment, which has been shown to exhibit a positive correlation with the expression of p21WAF1/CIP1 [34]. Therefore, we suspect that the decrease in PD-L1 expression observed in H460 and HCC827 cells may not be directly influenced by H3 and H4 acetylation and may be through another mechanism that could be influenced by p21WAF1/CIP1.

Similar to our results with lung cancer cells, the treatment of MCF7 human breast cancer cells with SAHA was also shown to induce the expression of p21WAF1/CIP1 as a consequence of the activation of two Sp1 sites located at −782 and −769 positions that are relative to the transcription start site of the p21WAF1/CIP1 gene. The Sp1 and Sp3 proteins are the main factors that typically bind to the Sp1 site of the p21WAF1/CIP1 promoter. However, it has been reported that SAHA did not alter DNA binding activities of Sp1 and Sp3 proteins, suggesting that the SAHA-mediated increase in p21WAF1/CIP1 promoter activity resulted from a mechanism other than altering the DNA binding activities of Sp1 and Sp3. In addition, SAHA-induced accumulation of acetylated histones in the chromatin of the p21WAF1/CIP1 gene was shown to be associated with an increase in p21WAF1/CIP1 expression in T24 bladder carcinoma cells. These findings further indicated that the induction of p21WAF1/CIP1 by SAHA could be regulated, at least in part, by the acetylation of the H3 and H4 histones that are associated with the p21WAF1/CIP1 gene [34]. Furthermore, the up-regulation of the p21WAF1/CIP1 protein expression was observed along with the down-regulated expression of the CDK family of proteins (CDK4 and CDK6) in both lung cancer cells. The decrease in the CDK4 and CDK6 levels correlated well with the decrease in the pRB levels following SAHA treatment. These results initially suggested that the elevation of p21WAF1/CIP1 must have reduced the levels of these cyclin-dependent kinases and their related phosphorylation activities, as evidenced by the decrease in pRB levels.

The PI3K/AKT signaling pathway represents a critical component in the process of cancer pathogenesis, primarily through the activation of downstream effectors that regulate cell survival, proliferation, and tumor angiogenesis [35,36,37,38]. Its role in modulating PD-L1 expression in cancer cells was initially suggested by the observation that the treatment of melanoma cells with a BRAF inhibitor causes a reduction in PD-L1 levels [39]. This was further supported by the findings that PTEN knockdown could result in PD-L1 up-regulation, an effect that was reversed by AKT inhibition [40,41]. Though the induction of transcription was shown to be the primary cause for increased PD-L1 expression following activation of the PI3K/AKT axis, post-translational mechanisms that can impact the stability of PD-L1 have also been suspected to be involved. For example, AKT activation in colon cancer cells led to the up-regulation of PD-L1 protein levels without increasing PD-L1 mRNA expression [41]. Therefore, it is speculated that alterations in the PI3K/AKT pathway could regulate PD-L1 expression by both transcriptional and post-transcriptional mechanisms in a cell- and tissue-specific manner [41]. While AKT inhibition resulted in a significant reduction of PD-L1 expression, its downstream effector mTOR/S6 was found not to be involved in AKT-induced regulation of PD-L1 expression [39,42]. On the other hand, NF-kB, which is a downstream target of AKT has been shown to transcriptionally regulate PD-L1 expression in cancer cells [43,44,45,46,47,48]. As an extension of the PI3K/AKT axis, the HIF-1α (hypoxia-inducible factor alpha) levels were also found to be associated with increased PD-L1 expression and reported to be one of the causes of the down-regulation of T-cell function [49,50,51,52,53,54,55]. This interesting correlation led to the suggestion that hypoxic environments can also result in immune suppression in addition to promoting angiogenesis, cell proliferation, and inhibition of apoptosis. It has been reported that HIF-1α can induce PD-L1 transcription through binding to the hypoxia response element of the PD-L1 promoter [55,56,57,58,59,60,61].

**Figure 10:**
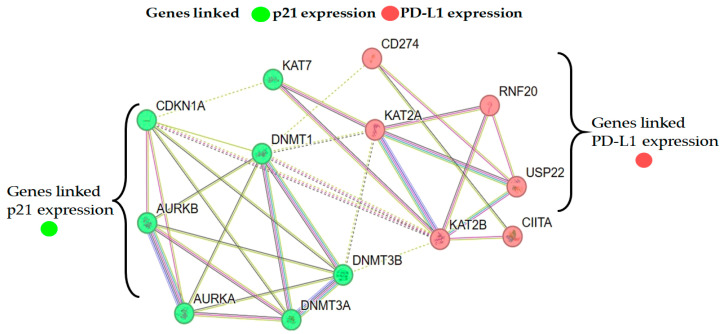
Network analysis of the H460 and HCC827 protein–protein interaction (PPI) genes that are known to be linked to the expression of PD-L1 and p21. Circles represent the genes (p21-linked genes are marked in green and PD-L1-linked genes are marked in red), and the connecting lines represent the number of interactions between them. The green color nodes are p21 linked genes and red color nodes are PD-L1 linked genes. The color of the edges are as described in the Network section of the Getting Started page of String Help (https://string-db.org/help/getting_started/) (accessed on 16 July 2025).

Furthermore, the PI3K pathway, particularly AKT, was shown to be inversely correlated to the p21WAF1/CIP1 levels in HCT116 colon cancer cells [62,63]. Therefore, we suspect that the significant elevation of p21 observed in the lung cancer cells treated with SAHA might have led to a decrease in the AKT levels, which in turn could have contributed to the decrease in PD-L1 expression. While our findings suggest potential involvement of the PI3K/AKT signaling axis in the regulation of PD-L1 and histone modifications, it should be noted that no pathway-specific experiments were conducted in this study. The PI3K/AKT pathway-related discussion is a mechanistic hypothesis supported by the prior literature rather than direct evidence from our study. Future experiments using pharmacological inhibitors or pathway reporters will determine the contribution of PI3K/AKT signaling to the change in PD-L1 levels. Another interesting result derived from our experiment that might have contributed to the down-regulation of PD-L1 expression is the decrease in CDK 4/6 levels. The same group had also shown that inhibition of CDK4/6 using palbociclib suppressed PD-L1 expression by blocking pRB phosphorylation and its dissociation from E2F1 [64,65]. In our experiments, we saw a significant reduction in the levels of pRB along with a decrease in the CDK 4/6 levels. Moreover, p21WAF1/CIP1 is also known to inhibit CDK 4/6, leading to the suppression of E2F-mediated transcriptional activity [66,67,68,69]. Therefore, we suspect that both p21WAF1/CIP1-mediated inhibition as well as the down-regulation of CDK 4/6 and pRB may majorly contribute to the decrease in PD-L1 expression in lung cancer cells following SAHA treatment. Additionally, STAT3 has been demonstrated to bind to the PD-L1 promoter to regulate PD-L1 transcription. While mutations of ALK (anaplastic lymphoma kinase) were reported to increase PD-L1 expression, treatment of cells with siRNA against STAT3 was able to abolish the ALK-mediated induction of PD-L1 [70,71,72,73,74]. In addition, the transfection of latent membrane protein-1 (LMP1) derived from EBV (Epstein–Barr Virus) was also shown to increase PD-L1 expression, with concomitant elevation of phosphorylated STAT3 (pSTAT3). On the other hand, the inhibition of pSTAT3 by the JAK3 inhibitor CP-690550 was shown to reduce LMP1-induced PD-L1 expression [75,76]. Thus, multiple pathways and their related transcriptional factors have been shown to regulate PD-L1 expression by STAT3 and thereby contribute to the evasion of cancer cells from the immune system. Unexpectedly, there was noticeable elevation of non-phosphorylated STAT3 in our experiments following SAHA treatment, which may be due to direct influence of the epigenetic alterations, such as histone hyperacetylation, on the transcription of the STAT3 gene. However, analyzing the status of pSTAT3 may indicate whether STAT3 has any role in regulating PD-L1 expression. The micro-array experiments suggest that some of the HDACs, such as 3, 5, 9, and 11, are elevated during SAHA treatment in H460 cells. However, in HCC827 cells, only HDACs 3, 5, and 9 were up-regulated. This may be some sort of rebound mechanism to overcome the strong inhibition of HDACs, because literature evidence indicates that SAHA was able to reduce the levels of HDAC2 and 4 in cancer cells [77]. Interestingly, HDAC10 alone was down-regulated in HCC827 cells, but there was no change observed in HDAC2 or HDAC4 in either cancer cell line. In addition, among the SET domain containing a family of proteins, SET1B, SETD6, and SETD7 were significantly down-regulated, suggesting a possible decrease in histone methylation. Similarly, SAHA treatment was able to induce interesting changes with other histone and protein methyl transferases also that might lead to decrease in methylations of lysine residues in histones and some of the arginine residues in key functional proteins. In addition, the decrease in DNMT3A and DNMT3B levels suggests there might be a reduction in the methylations of DNA following SAHA treatment. When there is increased acetylation due to HDAC inhibition, decreased methylations have been reported in the literature. This is probably part of transforming the heterochromatin to euchromatin following hyperacetylation. STRING network analysis revealed several hub genes with direct or indirect regulatory roles in PD-L1 expression, including USP22, DNMT1, RNF20, KAT2A, and CIITA. USP22 is known to stabilize PD-L1 through deubiquitination [78], while DNMT1 contributes to PD-L1 promoter methylation and transcriptional silencing [79,80]. RNF20, a key regulator of histone H2B ubiquitination, has been linked to PD-L1 transcriptional control [81], and KAT2A-mediated histone acetylation promotes PD-L1 expression through enhanced chromatin accessibility [82,83]. CIITA, a transcriptional activator of MHC class II genes, has also been implicated in the broader regulation of immune checkpoint signaling, including the impact on PD-L1 levels [84]. In addition, AURKA, AURKB, CIITA, DNMT1, DNMT3A, DNMT3B, KAT2A, KAT7, and USP22 levels were also significantly down-regulated while RNF20 and KAT2B were up-regulated in H460 and HCC827 cells, which may be due to the influence of the hyper-acetylation of histones caused by SAHA treatment (Figure 10, Table 4). These findings suggest that the treatment-induced reduction in PD-L1 expression may, at least in part, be mediated by alterations in the activity of these hub genes, thereby linking our transcriptomic network results with the immune checkpoint status. Thus, in the current study, through dose-dependent treatment, it has been confirmed that SAHA decreases the level of PD-L1 while p53 and p21WAF1/CIP1 levels are elevated, and it showed a negative correlation coefficient of −0.885 when the analysis was conducted using the band intensities. The p21WAF1/CIP1 changes observed in our experiments can also be correlated to the decrease in CDK4/CDK6 and pRB levels, which are known to decrease the expression of PD-L1. Though the increase in p21WAF1/CIP1 levels correlate well with the decrease in PD-L1 expression, the specific knockdown of this suspected mediator gene using siRNA or CRISPR/Cas9, followed by PD-L1 expression analysis at both mRNA and protein levels, would confirm the inter-relationship.

Specifically, our findings suggest that the modulation of p21 and the associated PD-L1 expression changes could be leveraged in rational combination strategies with immune checkpoint inhibitors. For example, integrating epigenetic modulators that impact p21/PD-L1 regulation resulting in reduced PD-1 blockade may enhance antitumor immunity, particularly in tumors that exhibit intrinsic resistance to checkpoint monotherapy. Although further validation for some of the findings is essential, these insights could help with the design of biomarker-driven combination immunotherapy strategies in the future.

## 5. Conclusions

**Figure 11:**
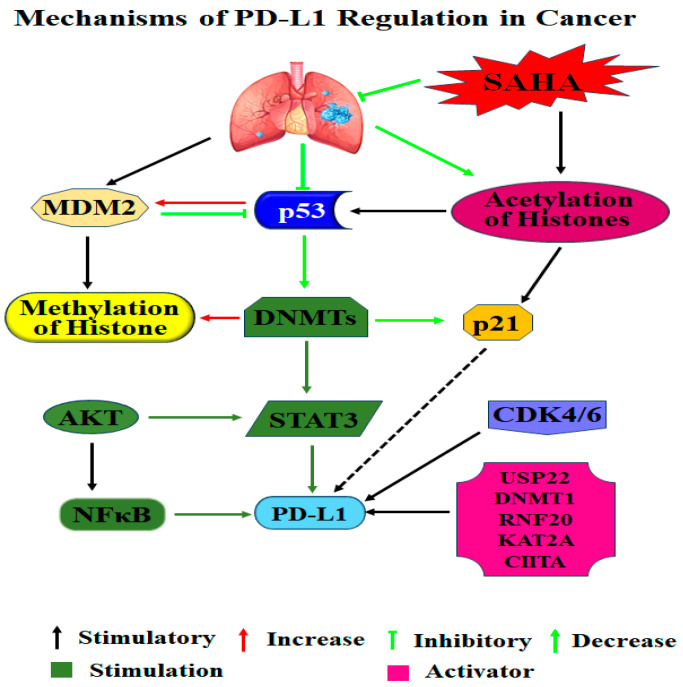
Mechanisms of PD-L1 regulation in lung cancer. PD-L1 expression in tumor cells is regulated at multiple levels. The p53–p21 axis can negatively influence PD-L1 transcription, while oncogenic pathways such as PI3K/AKT and JAK/STAT promote its up-regulation. Epigenetic mechanisms play a critical role: histone acetylation and methylation dynamically regulate chromatin accessibility, and DNA methylation (e.g., via DNMT1) can repress or activate PD-L1 transcription depending on context. Collectively, these mechanisms contribute to PD-L1-mediated immune evasion and tumor survival.

In conclusion, the use of HDAC inhibitors for treating different cancers started more than a decade ago. However, augmenting immunotherapy by decreasing the levels of PD-L1 in target cells is a new phenomenon that is proven to be a viable approach in our experiments. Even though identifying the exact mechanism that is responsible for the down-regulation of PD-L1 following SAHA treatment is yet to be established, our study results suggest that the immunotherapeutic outcomes of the check point inhibitors can be enhanced by combining with HDAC inhibitors such as SAHA. The epigenetic modifier SAHA can unwind chromatin structures and recruit “acetyl-lysine readers” to the acetylated sites that can subsequently trigger downstream target gene expression. HDAC inhibition has been described to modify gene transcription by increasing acetylation of histones, and H2A at lysine 20, H3 at Lysine 9 (H3K9ac), Lysine 14 (H3K14ac), or lysine 27 (H3K27ac) and H4 at Lysine 12 in the promoter regions [18,85,86,87,88]. Since SAHA is one of the most powerful HDACIs, it was of interest to consider the alterations in the PD-L1 expression in relation to the acetylation of histone over time with continued treatment. During our experiments SAHA was able to block PD-L1 expression in a dose-dependent manner. Also, the p21WAF1/CIP1-mediated inhibition of CDK 4/6 and pRB seems to contribute to the down-regulation of PD-L1 expression. So far, various clinical studies have demonstrated that blocking the PD-1/PD-L1 interaction, which is a vital regulator of T-cell activity, could boost the antitumor immune response and thereby inhibit the growth of the tumors. However, a clear understanding of the molecular and cellular events that can influence the levels of PD-L1 has been lacking [89,90,91,92]. To further advance the knowledge related to PD-L1-mediated immune escape, the impact of the HDAC inhibitors on the expression levels of PD-L1 was systematically analyzed in his study using the H460 and HCC827 lung cancer cells (Figure 11). Our results clearly indicate that the inhibition of HDACs using SAHA produces significant down-regulation of PD-L1 in the lung cancer cells. As we anticipated the SAHA treatment decreased the expression of PD-L1 indirectly through elevation of p21WAF1/CIP1 in our experimental model. The results obtained using the in vitro model require further validation using in vivo studies.

## Figures and Tables

**Table 1:** List of the Antibodies.

Primary Antibodies	Vendor	Catalog No	WB/IF
PD-L1	R&D Systems	AF1019	1:1000
MDM2	CST	sc-13161	2:1000
p53	CST	32532	1:1000
pp53 (Phospho-p53)	CST	12571	1:1000
p21	CST	2947	1:1000
p27	CST	3686	1:1000
STAT3	CST	9139	1:1000
pSTAT3 (Phospho-Stat3)	CST	9145	1:1000
NFkB	CST	4764	1:1000
CDK4	CST	12790	1:1000
CDK6	CST	13331	1:1000
pRB (Phospho-RB)	CST	8516	1:1000
Ac-H2A (Acetyl Histone H2A)	CST	2576	1:1000
Ac-H2B (Acetyl Histone H2B)	CST	34156	1:1000
Ac-H3 (Acetyl Histone H3)	CST	9677	1:1000/1:1500
Ac-H4 (Acetyl Histone H4)	CST	13944	1:1000/1:1500
Tri Me-H3 (Tri-Methyl Histone H3)	CST	4909	1:1000
Tri Me-H4 (Tri-Methyl Histone H4)	CST	5737	1:1000
DNMT3B	CST	67259	1:1000
MGMT	CST	2739	1:1000

**Table 2:** Changes in expression for cancer-related human epigenetic alteration genes between control and SAHA in lung cancer cells. The table lists genes from the experiment in Figure 8, which exhibit at least a three-fold or greater difference in expression between untreated and SAHA-treated (**A**) H460 and (**B**) HCC827.

**(A) H460—Up- and Down-Regulated Genes**			
**Gene**	**Description**	**Fold Change**	**Accession Number**
HDAC9	Histone deacetylase 9	9.03	NM_178425
HDAC3	Histone deacetylase 3	3.96	NM_003883
NCOA3	Nuclear receptor coactivator 3	3.92	NM_181659
HDAC5	Histone deacetylase 5	3.68	NM_005474
KDM5B	Lysine (K)-specific demethylase 5B	3.06	NM_006618
KMT2C	Myeloid/lymphoid or mixed-lineage leukemia 3	3.02	NM_170606
DZIP3	DAZ interacting protein 3, zinc finger	2.54	NM_014648
HDAC11	Histone deacetylase 11	2.42	NM_024827
SETDB2	SET domain, bifurcated 2	2.42	NM_031915
RNF20	Ring finger protein 20	2.08	NM_019592
AURKA	Aurora kinase A	−5.21	NM_003600
SETD7	SET domain containing (lysine methyltransferase) 7	−3.93	NM_030648
DNMT3A	DNA (cytosine-5-)-methyltransferase 3 alpha	−3.92	NM_022552
NSD1	Nuclear receptor binding SET domain protein 1	−3.50	NM_022455
HDAC7	Histone deacetylase 7	−3.21	NM_001098416
AURKB	Aurora kinase B	−2.75	NM_004217
SUV39H1	Suppressor of variegation 3–9 homolog 1 (Drosophila)	−2.74	NM_003173
WHSC1	Wolf-Hirschhorn syndrome candidate 1	−2.71	NM_007331
SMYD3	SET and MYND domain containing 3	−2.69	NM_022743
SETD6	SET domain containing 6	−2.55	NM_024860
SETD1B	SET domain containing 1B	−2.38	NM_015048
KAT6B	K(lysine) acetyltransferase 6B	−2.33	NM_012330
PRMT1	Protein arginine methyltransferase 1	−2.23	NM_001536
KAT2A	K(lysine) acetyl transferase 2A	−2.09	NM_021078
KAT5	K(lysine) acetyltransferase 5	−2.01	NM_006388
USP21	Ubiquitin-specific peptidase 21	−2.01	NM_012475
**(B) HCC827—Up- and Down-Regulated Genes**			
**Gene**	**Description**	**Fold Change**	**Accession Number**
HDAC3	Histone deacetylase 3	7.05	NM_003883
KDM5C	Lysine (K)-specific demethylase 5C	4.67	NM_004187
KMT2A	Myeloid/lymphoid or mixed-lineage leukemia (trithorax homolog, Drosophila)	4.11	NM_005933
KMT2C	Myeloid/lymphoid or mixed-lineage leukemia 3	4.09	NM_170606
KDM6B	Lysine (K)-specific demethylase 6B	3.48	NM_001080424
HDAC9	Histone deacetylase 9	2.90	NM_178425
RNF20	Ring finger protein 20	2.25	NM_019592
NCOA3	Nuclear receptor coactivator 3	2.14	NM_181659
DNMT3B	DNA (cytosine-5-)-methyltransferase 3 beta	−21.62	NM_006892
NSD1	Nuclear receptor binding SET domain protein 1	−6.06	NM_022455
AURKB	Aurora Kinase B	−4.65	NM_004217
DNMT3A	DNA (cytosine-5-)-methyltransferase 3 alpha	−3.79	NM_022552
NEK6	NIMA (never in mitosis gene a)-related kinase 6	−3.62	NM_014397
KAT6B	K(lysine) acetyltransferase 6B	−3.22	NM_012330
SUV39H1	Suppressor of variegation 3–9 homolog 1 (Drosophila)	−2.82	NM_003173
PRMT1	Protein arginine methyltransferase 1	−2.62	NM_001536
WHSC1	Wolf-Hirschhorn syndrome candidate 1	−2.59	NM_007331
AURKA	Aurora kinase A	−2.51	NM_003600
SETD1B	SET domain containing 1B	−2.29	NM_015048
USP21	Ubiquitin-specific peptidase 21	−2.22	NM_012475
HDAC10	Histone deacetylase 10	−2.19	NM_032019
RPS6KA3	Ribosomal protein S6 kinase, 90kDa, polypeptide 3	−2.18	NM_004586

**Table 3:** These indicate the number of clusters in (**A**) H460 and (**B**) HCC827 cells and lists of up- and down-regulated genes in the clusters.

**(A)**		**Gene Count**	**Protein Names**
Clusters 1	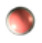	12	KDM5B, KMT2C, NSD1, NSD2, ASH1L, PRMT1, RNF20, SETD1B, SETD6, SETD7, SETDB2, SMYD3
Clusters 2	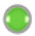	11	DNMT3A, HDAC11, HDAC3, HDAC5, HDAC7, HDAC9, KAT2A, KAT5, KAT6B, NCOA3, SUV39H1
Clusters 3	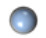	3	AURKA, AURKB, USP21
**(B)**		**Gene Count**	**Protein Names**
Clusters 1	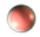	15	AURKB, DNMT3A, DNMT3B, HDAC10, HDAC3, HDAC9, KAT6B, KDM5C, KDM6B, KMT2A, NSD1, NSD2, NCOA3, PRMT1, SUV39H1
Clusters 2	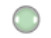	2	AURKA, USP21
Clusters 3	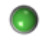	2	RNF20, SETD1B
Clusters 4	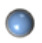	1	KMT2C

**Table 4:** List of the genes that are known to be linked to the expression of p21 and PD-L1.

	p21- and PD-L1-Linked Genes
p21	AURKA, AURKB, DNMT3A, DNMT3B, DNMT1, KAT7, KAT2B
PD-L1	USP22, DNMT1, RNF20, KAT2A, CIITA

## Data Availability

The data presented in this study are available on request from the corresponding author due to time required for retrieval from the archive.

## Supplementary Materials

The following supporting information can be downloaded at: https://www.mdpi.com/article/10.3390/cancers17172919/s1, Figure S1: Uncropped Western Blot.

## Abbreviations

The following abbreviations are used in this manuscript:

Ac-H2A	Acetyl Histone H2A
Ac-H2B	Acetyl Histone H2B
Ac-H3	Acetyl Histone H3
Ac-H4	Acetyl Histone H4
ALK	Anaplastic Lymphoma Kinase
ATTC	American Type Culture Collection
BC	Breast Cancer
BCA	Bicinchoninic Acid
BSA	Bovine Serum Albumin
CTLA-4	Cytotoxic T-Lymphocyte-Associated Protein 4
CD80	B7-1
DNMTs	DNA Methyltransferases
∆∆Ct	Double Delta Ct
EBV	Epstein–Barr Virus
FBS	Fetal Bovine Serum
FDR	False Discovery Rate
GC	Gastric Cancer
HATs	Histone Acetyltransferases
HDACIs	Histone Deacetylase Inhibitors
HC	Hodgkin’s Lymphoma
HCC	Hepatocellular Carcinoma
HIF-1α	Hypoxia-Inducible Factor Alpha
HNSCC	Head and Neck Squamous Cell Carcinoma
HRP	Horseradish Peroxidase
IC	Immune Checkpoint
ICC	Immunocytochemistry
MCC	Merkel cell carcinoma
MGMT	O-6-methylguanine-DNA methyltransferase
MLL1	Mixed-Lineage Leukemia Protein
NSCLC	Non-Small Cell Lung Cancer
PBS	Phosphate-Buffered Saline
PD-L1	Programmed Death Ligand-1
PPI	Protein–Protein Interaction
pp53	Phospho-p53
pRB	Phospho-RB
pSTAT3	Phosphorylated STAT3
PMSF	Phenyl Methyl Sulfonyl Fluoride
qRT-PCR	Quantitative Reverse Transcription PCR
RCC	Renal Cell Carcinoma
RPMI	Roswell Park Memorial Institute
SAHA	Suberoylanilide Hydroxamic Acid
TCC	Transitional Cell Carcinoma
Tri Me-H3	Tri-Methyl Histone H3
Tri Me-H4	Tri-Methyl Histone H4
