# Supplementary material for: Regulation of PD-L1 Expression by SAHA-Mediated Histone Deacetylase Inhibition in Lung Cancer Cells
Source: Cancers (Basel). 2025 Sep 5;17(17):2919. doi: 10.3390/cancers17172919 (PMC12428147; doi:10.3390/cancers17172919)

Regulation of PD-L1 Expression Through Histone Deacetylase Inhibition in Lung Cancer Cells

Notes for the image reviewers:

1. **Our lab cuts the membrane into two portions and develops the portions (High M. Wt & Low M. Wt) with different antibodies**
2. **The protein separation (mobility) varies due to percentage of gel used.**
3. **The protein of interest was identified based on the M.Wt shown by the antibody provider.**

Supplementary Figure S1 Uncropped Western Blot

Figure 1.

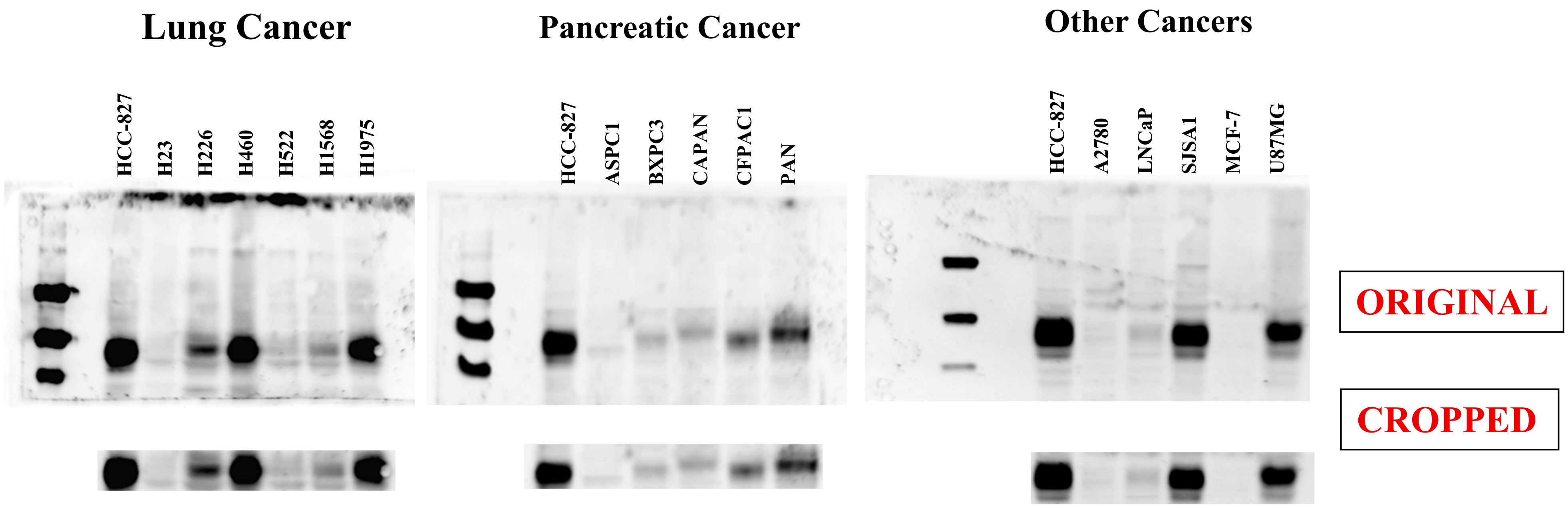

Figure 2.

ORIGINAL

CROPPED

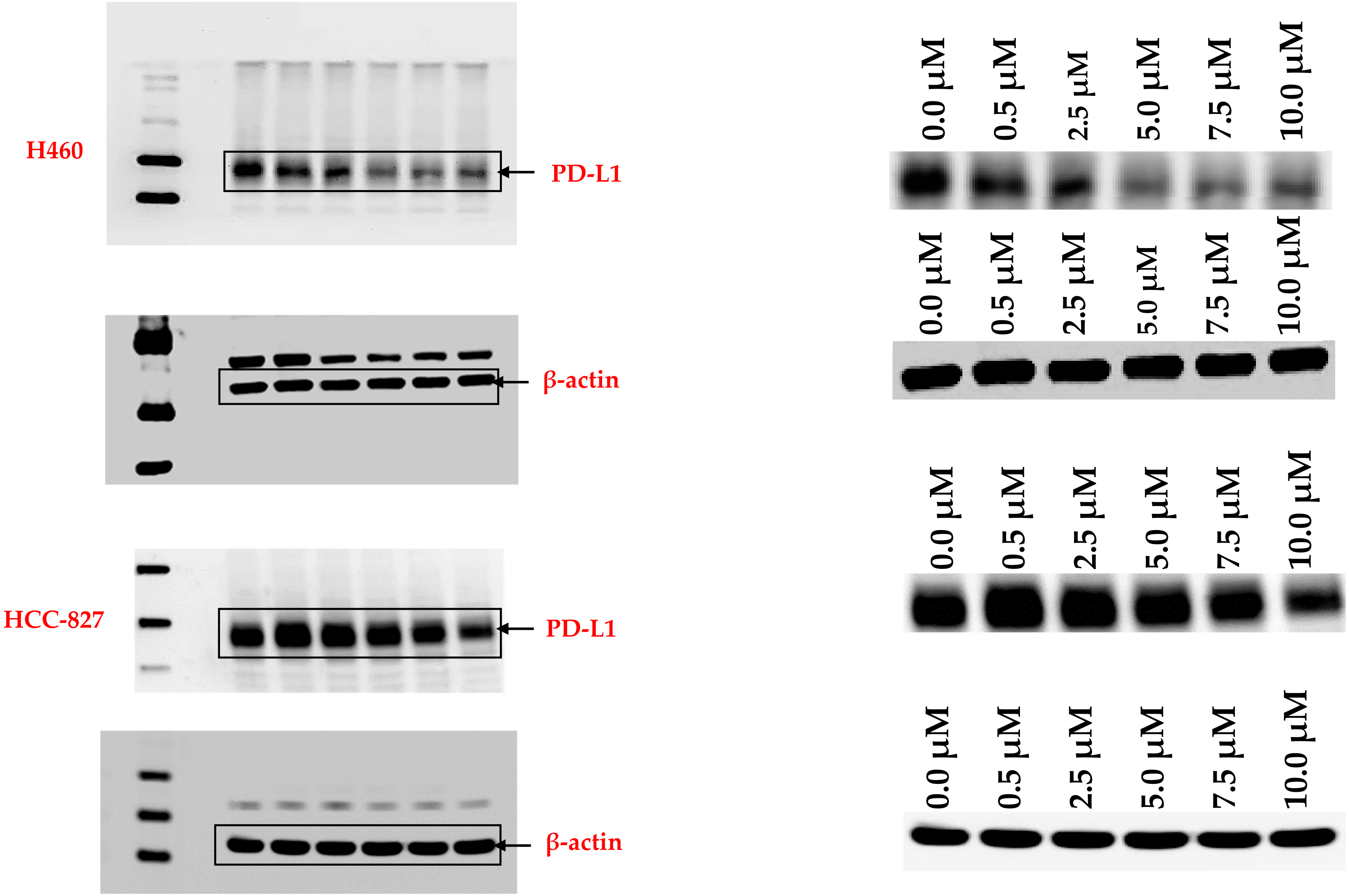

Figure 3A.

ORIGINAL | CROPPED

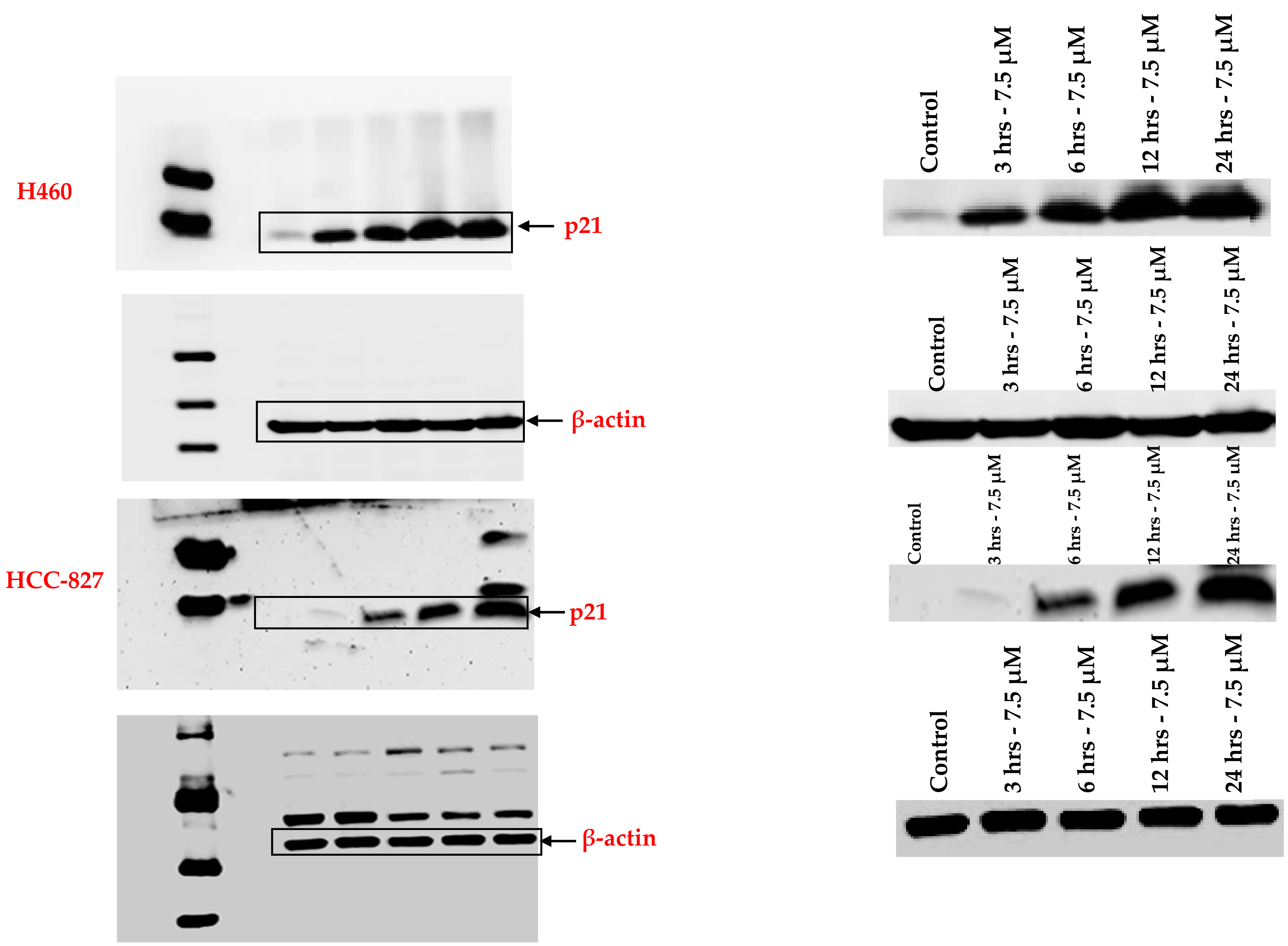

Figure 3B.

H460

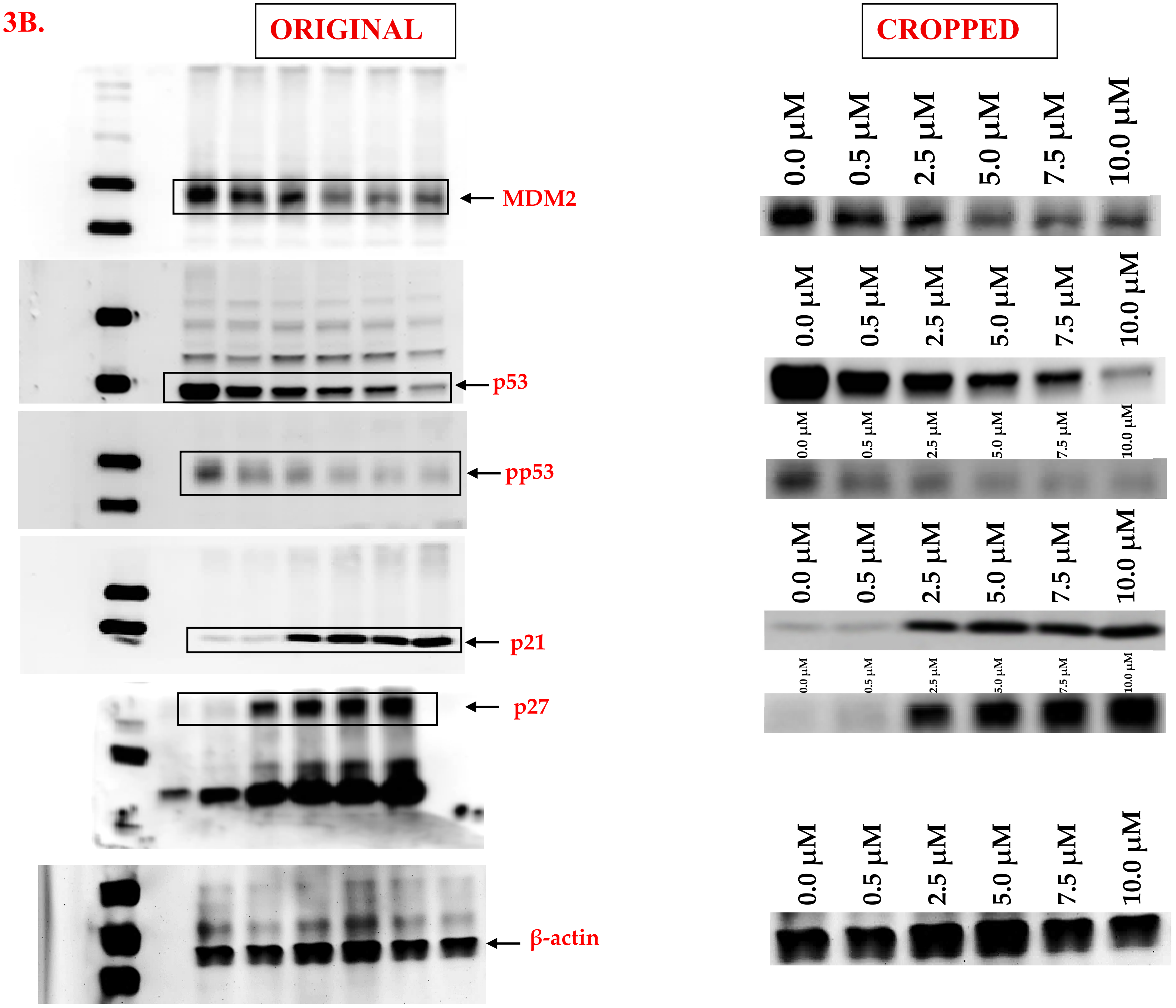

Figure 3C.

HCC827

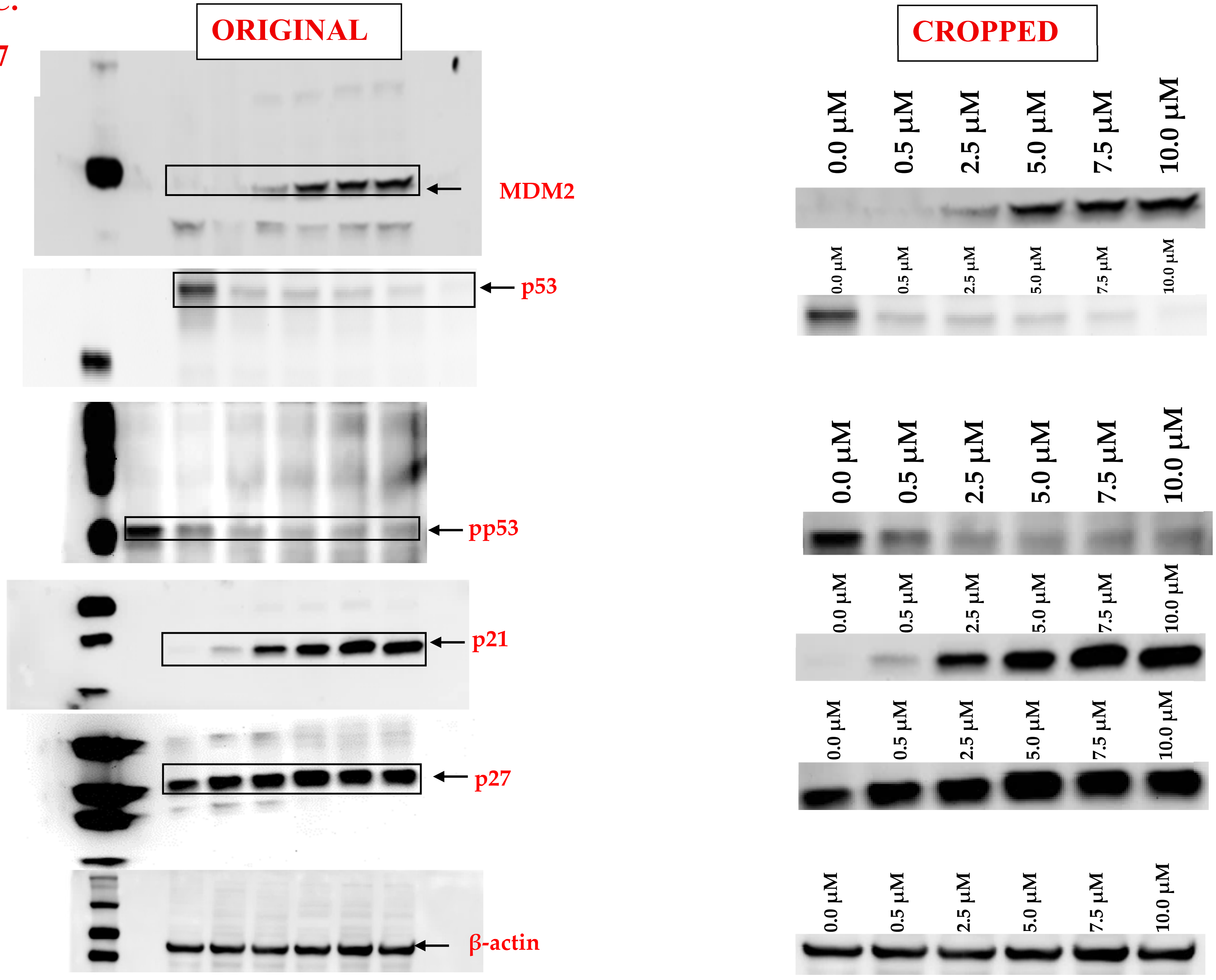

Figure 4A.

H460

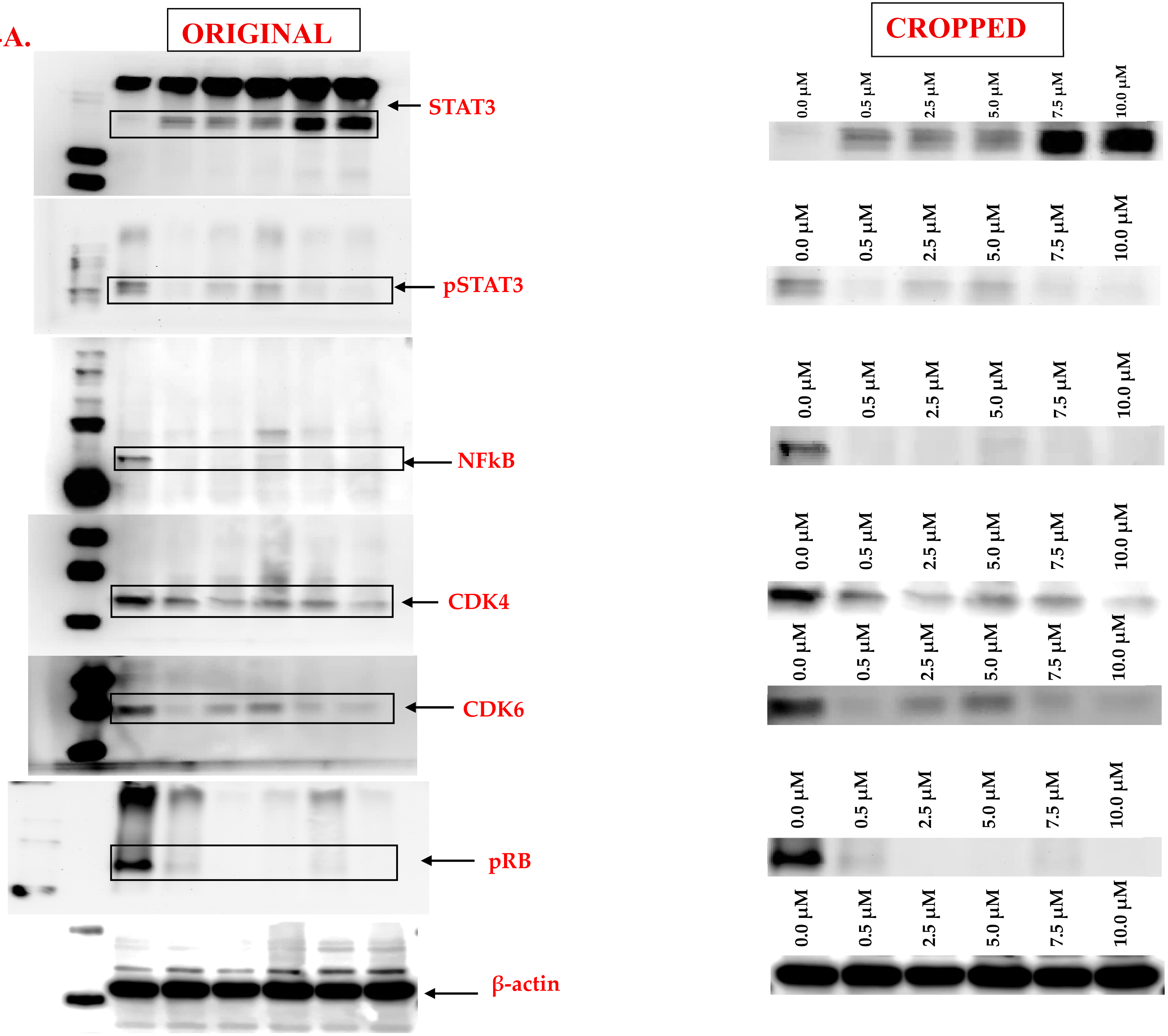

Figure 4B.

HCC827

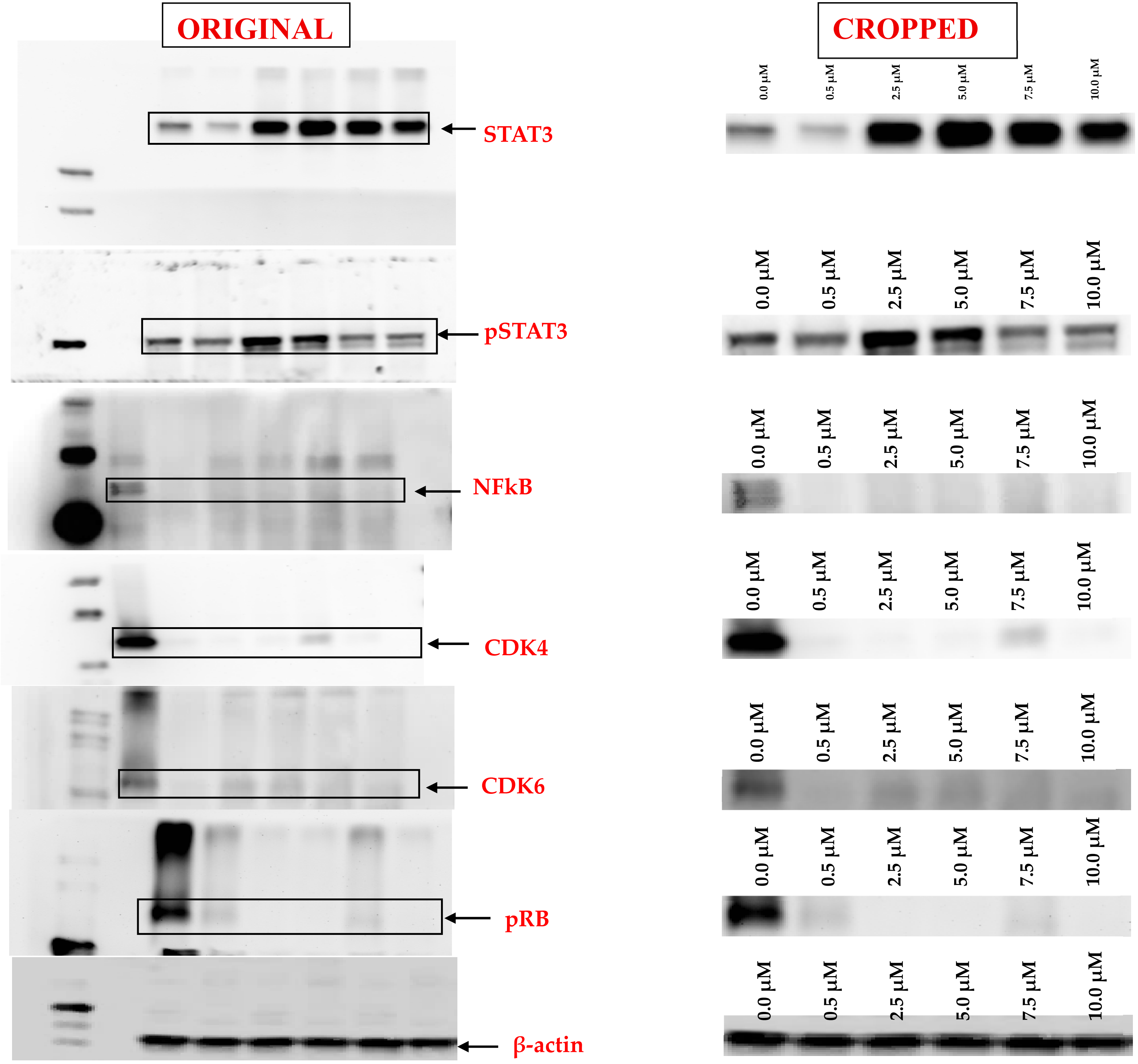

Figure 5A.

ORIGINAL

CROPPED

H460

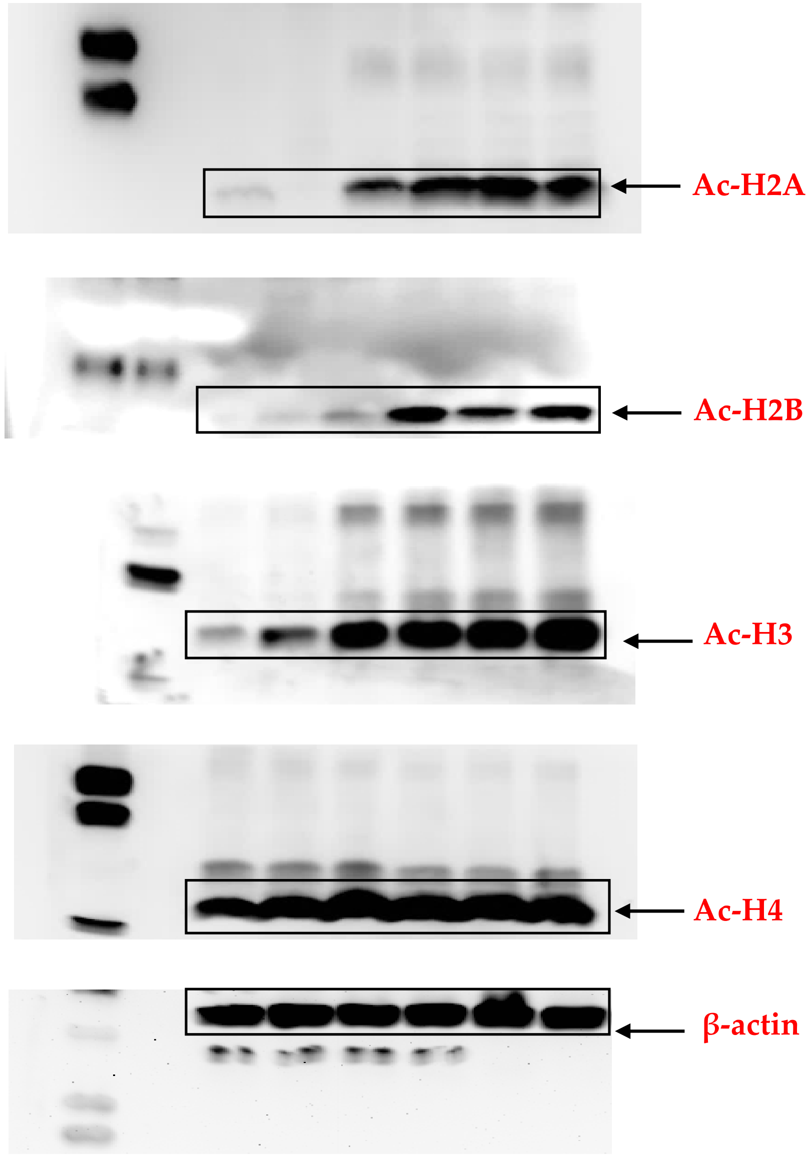

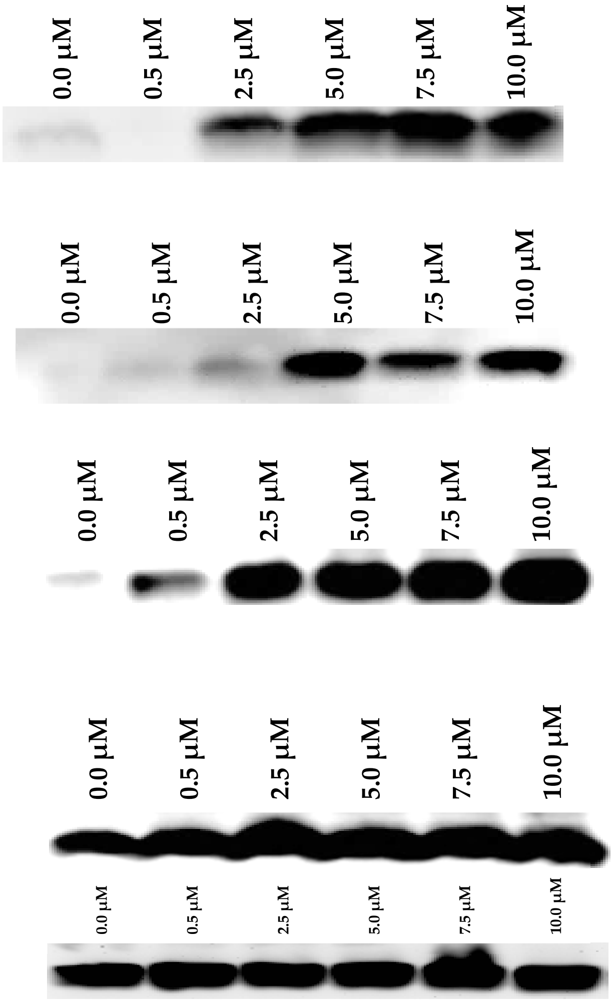

Figure 5B.

HCC827

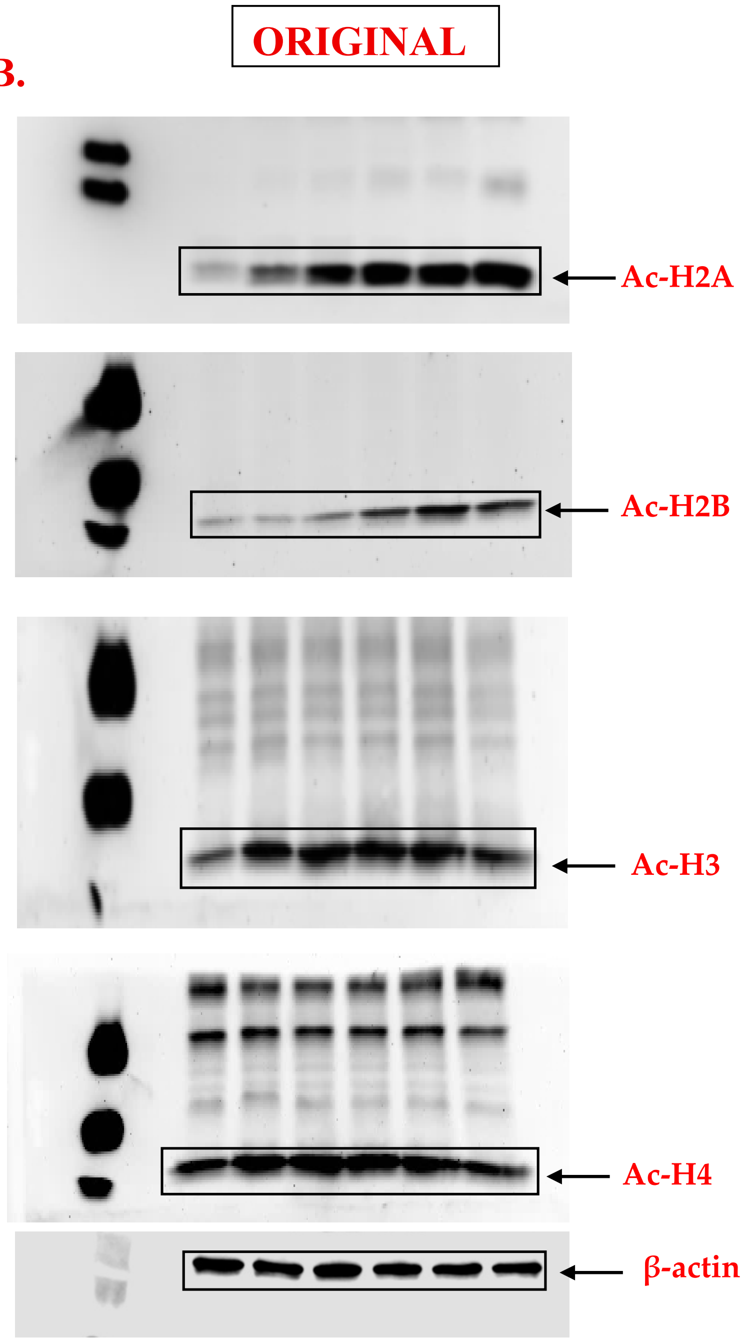

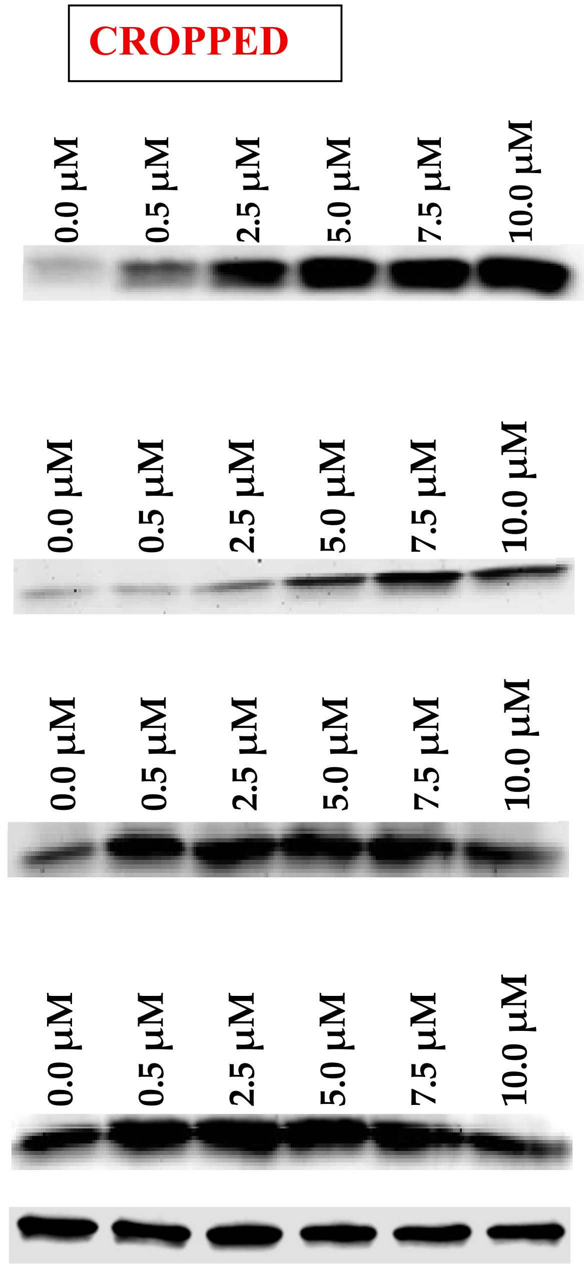

Figure 7A.

H460

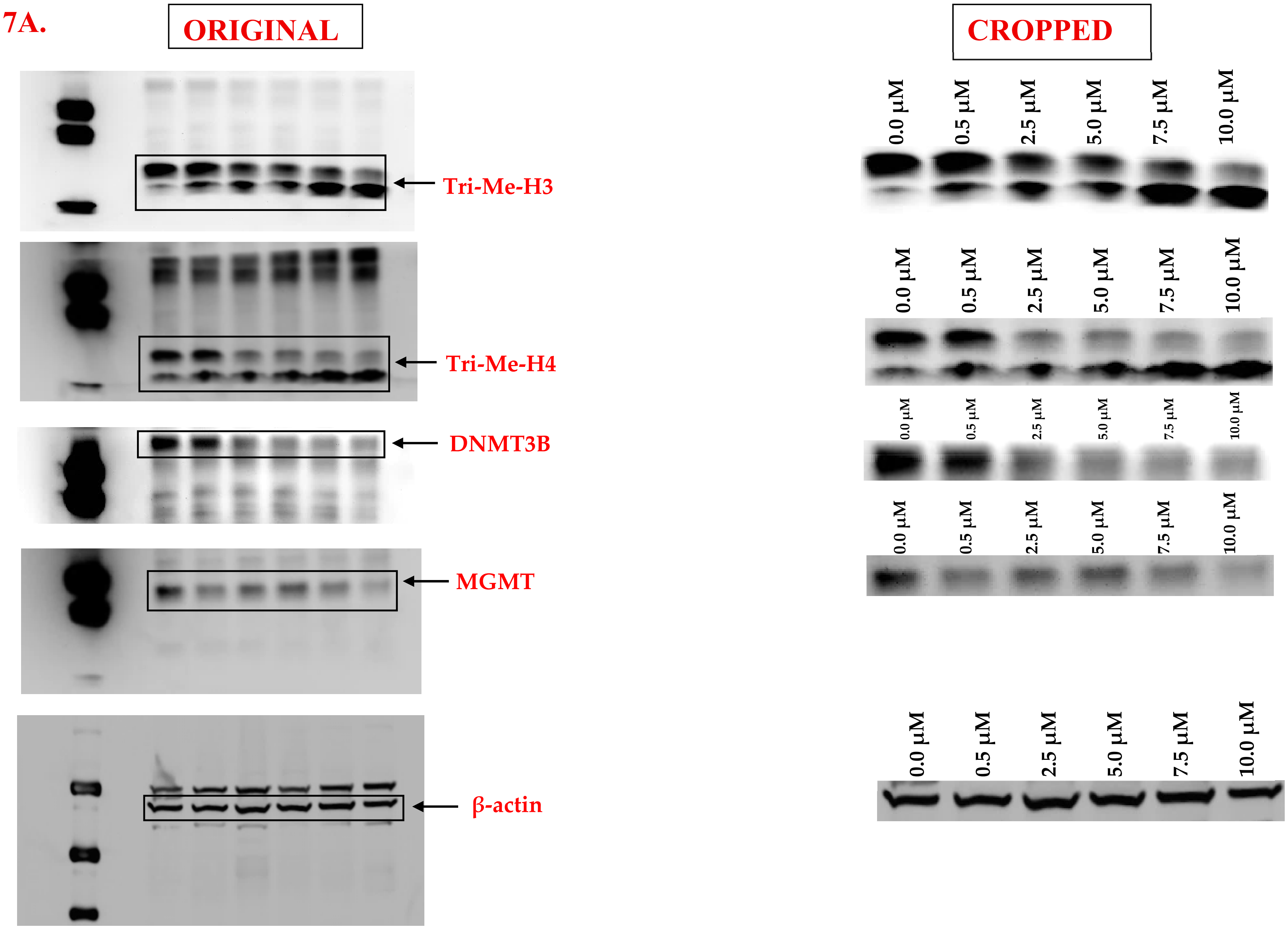

Figure 7B.

HCC827

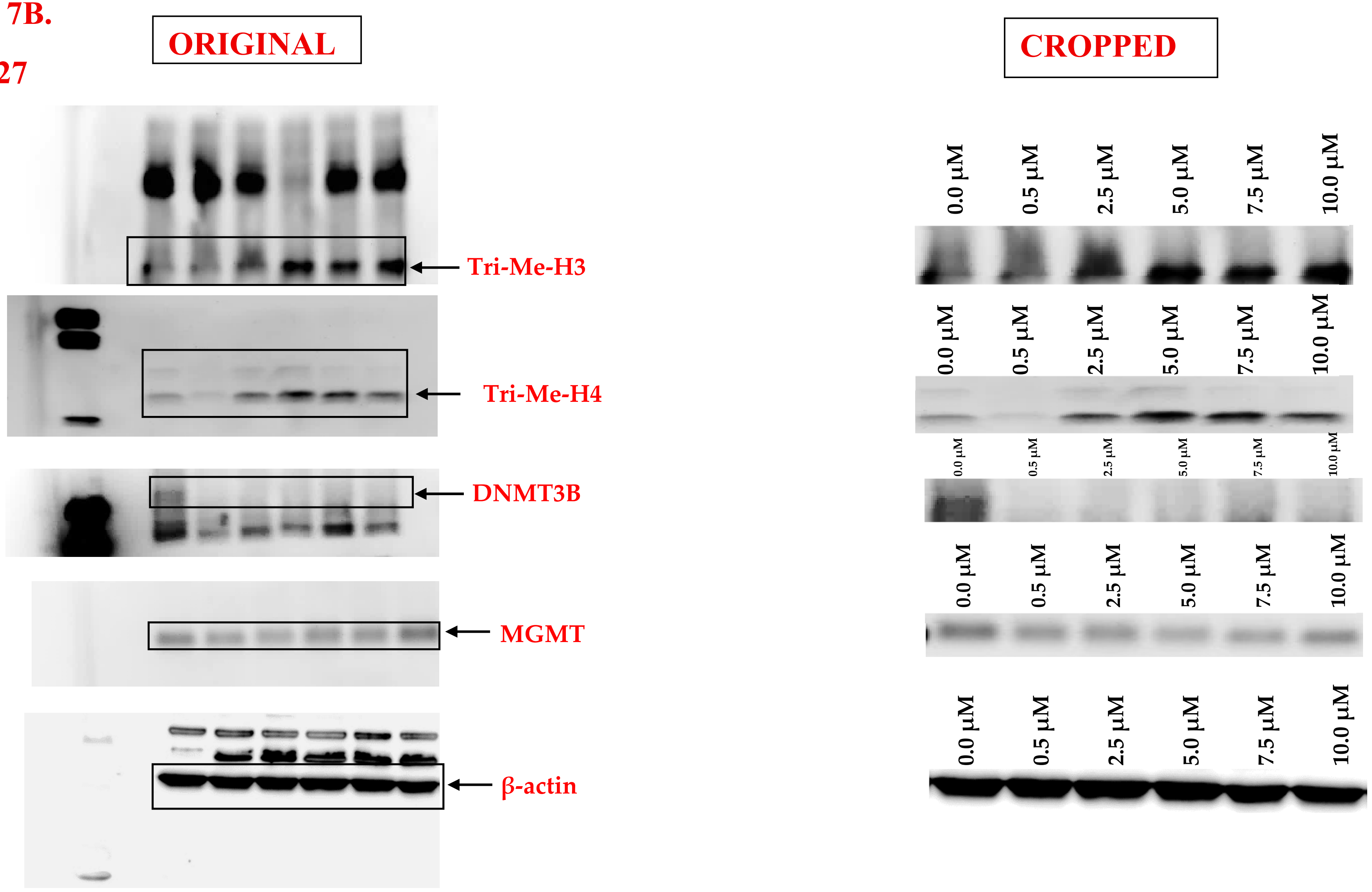

Supplement: Supplementary file 1 [file cancers-17-02919-s001.zip › cancers-3792834-supplementary.pdf]
